# LZTR1 regulates epithelial MHC-I expression via NF-κB1 to modulate CD8^+^ T cells activation

**DOI:** 10.1038/s41421-025-00837-6

**Published:** 2025-10-29

**Authors:** Rundong Jiang, Zhiqin Fang, Yutong Wang, Bo Huang, Junkun Liu, Lam C. Tsoi, Rachael Bogle, Zongbo Zhang, Yehong Kuang, Xin Li, Liang Dong, Liping Jin, Johann E. Gudjonsson, Mingzhu Yin, Xiang Chen

**Affiliations:** 1 Department of Dermatology, Hunan Engineering Research Center of Skin Health and Disease, Hunan Key Laboratory of Skin Cancer and Psoriasis, Xiangya Hospital, Central South University, Changsha, Hunan China; 2 National Engineering Research Center of Personalized Diagnostic and Therapeutic Technology, Central South University, Changsha, Hunan China; 3 Department of Dermatology, University of Michigan, Ann Arbor, MI USA; 4 Clinical Medicine Eight-Year Program, Xiangya School of Medicine, Central South University, Changsha, Hunan China; 5 Clinical Research Center, Medical Pathology Center, Cancer Early Detection and Treatment Center and Translational Medicine Research Center, Chongqing University Three Gorges Hospital, Chongqing University, Wanzhou, Chongqing China

**Keywords:** Autoimmunity, Mechanisms of disease, Transcription

## Abstract

The role of CD8^+^ tissue-resident memory T (CD8^+^ T_RM_) in inflammation is well established. However, the mechanisms by which CD8^+^ T_RM_ cells are activated in tissues have remained elusive. Here, we show that Leucine zipper-like transcription regulator 1 (LZTR1), a substrate adaptor for cullin3 (CUL3) ubiquitin ligase complex, regulates CD8^+^ T_RM_ activation and proliferation in cutaneous and colonic epithelia through modulation of major histocompatibility complex class I (MHC-I) expression in an NF-κB1-dependent manner. Mechanistically, LZTR1 modulates MHC-I transcription by regulating co-translational biogenesis of NF-κB1 (p50) in a ubiquitination-independent but proteasome-dependent manner through direct binding with ribosome and proteasome. Loss of LZTR1 leads to suppression of CD8^+^ T_RM_ activation and proliferation and decreased production of IL-17A with blunting of inflammatory responses in both cutaneous and gut epithelia in vivo. In summary, these data identify LZTR1 as a novel regulator of CD8^+^ T_RM_ function and provide insights into the mechanisms that drive and maintain CD8^+^ T-cell responses in epithelial-associated autoimmune diseases.

## Introduction

Despite the advent of highly effective targeted biologics against pro-inflammatory cytokines such as tumor necrosis factor (TNF) and interleukin-17 (IL-17) in the treatment of rheumatoid arthritis, spondylarthritis, and psoriasis, major challenges still remain, including incomplete response in some patients, recurrence upon cessation of treatment and loss of response over time^1–6^, providing a strong argument for the need of deeper understanding of disease mechanisms in autoimmune diseases. Recent studies have indicated the pathogenic role of CD8^+^ tissue-resident memory T (T_RM_) in immune-mediated inflammatory disorders, including psoriasis^7^, inflammatory bowel disease (IBD)^8^, and multiple sclerosis^9^, in which IL-17-producing CD8^+^ T_RM_ (CD8^+^ T_RM17_) rather than traditionally emphasized IL-17-producing CD4^+^ T_RM_ (CD4^+^ T_RM17_)^10,11^, drive disease progression and recurrence. Intriguingly, although both CD4^+^ and CD8^+^ T_RM17_ cells are present in psoriatic skin, their spatial distribution differs markedly, reflecting underlying heterogeneity in their activation triggers and interactions with antigen-presenting cells. In contrast to CD4^+^ T_RM_ cells, predominantly residing in dermis^11^, CD8^+^CD69^+^CD103^+^ T_RM_ cells are primarily enriched in the epidermis of inflamed psoriatic skin^12^. However, the precise triggers underlying CD8⁺ T_RM_ activation in autoimmune diseases remain largely undefined. In particular, the nature and drivers of the crosstalk between CD8^+^ T_RM_ cells and their target cells, as well as how this interaction reshapes the local microenvironment to promote inflammation, are still poorly understood^13,14^.

MHC class I (MHC-I) molecules play a major role in the immune system through presentation of endogenous peptide antigens to T-cell receptors (TCR) on CD8^+^ T cells and have been shown to play key roles in diseases such as psoriasis, ankylosing spondylitis and IBD^15–19^. Notably, HLA-Cw06:02 is a major risk allele for the development of psoriasis, along with contributions from antigens presented by HLA-A and HLA-B^20–22^. However, our understanding of the factors that regulate the expression of MHC-I and how epithelial and stromal cells influence CD8^+^ T-cell responses through MHC-I during initiation and reactivation of inflammatory responses remains limited.

Leucine zipper-like transcription regulator 1 (LZTR1), a substrate adaptor for cullin3 (CUL3) ubiquitin ligase complex, has been reported as a regulator of RAS/MAPK signaling to control cancer progression^23,24^, and as a candidate oncogene in melanoma^25^, indicating the heterogeneity of LZTR1 function in different disease conditions. Here, our findings uncover a unique molecular chaperone role of LZTR1 in enhancing the crosstalk between inflammatory CD8^+^ T_RM_ cells and epithelial cells through regulation of MHC-I expression and autoantigen presentation, exacerbating autoimmune reactions in the epithelium of both skin and gut.

## Results

### CD8^+^ T_RM17_ is a major source of IL17A in psoriatic epidermis

**Fig. 1:**
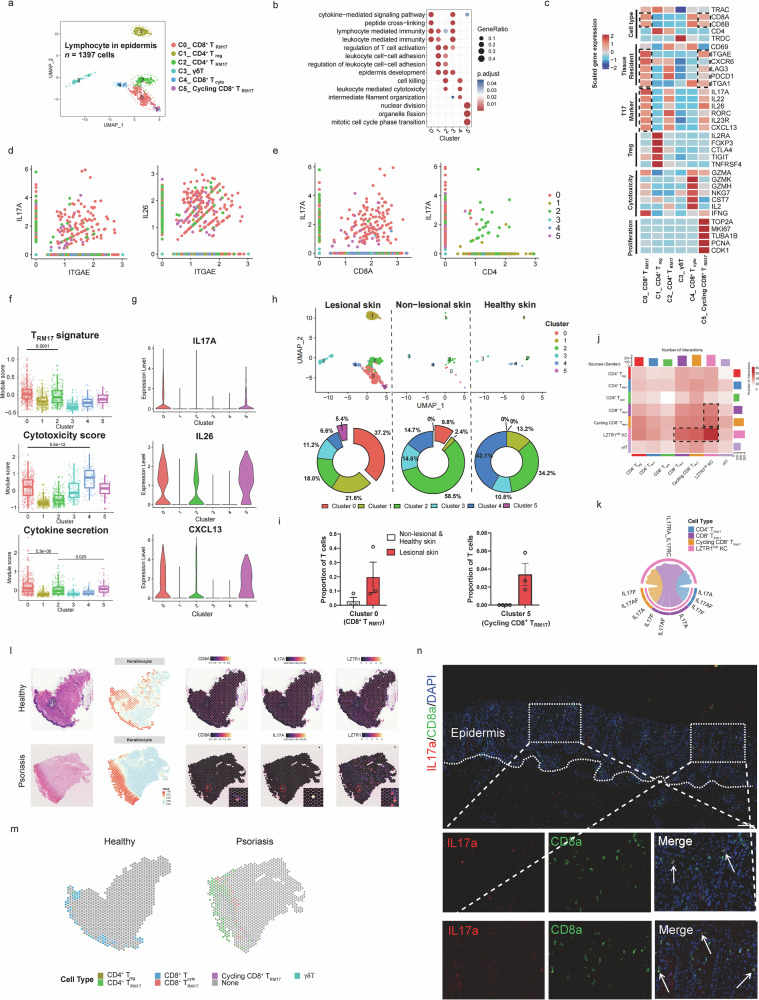
CD8^+^ T_RM17_ is a major source of IL17A in psoriatic epidermis. **a** UMAP plot visualizing 6 lymphocyte clusters in psoriasis epidermal immune microenvironment. **b** Dot plot showing GO biological process (BP) terms enriched in each cluster. **c** Heatmap of marker genes in 6 lymphocyte clusters. **d**, **e** Scatter plot showing co-expression of gene pairs. The color represents the sub-cluster identity of the cells. **f** Box plot showing the module scores in the lymphocyte subtypes. **g** Violin plot showing the expression of genes split by subtype. Each dot represents the gene’s expression in a single cell. **h** UMAP plot and percentage of each lymphocyte cluster in lesional skin, non-lesional skin, and healthy skin. **i** Changes in selected subpopulation composition in lesional and non-lesional group. **j** Heatmap showing the number of ligand-receptor pairs in lesional epidermis among the lymphocytes and *LZTR1*^*high*^ KCs. **k** Chord plot showing the IL17 family cytokines and respective receptor interactions between the lymphocyte subtypes and *LZTR1*^*high*^ KCs. **l** Representative H&E staining (left panel), spatial plot colored by cell-type (middle panel) and spatial transcriptome images of *CD8A*, *IL17A*, and *LZTR1* and expression (right panel) in normal and psoriatic human skin. **m** Scatter pie plot showing the distribution of the lymphocyte subtypes identified in scRNA-seq. **n** IF staining images of IL17A-producing CD8^+^ T cells in epidermis of psoriasis lesional skins.

Whereas the role of the innate and adaptive immune system in skin has been well studied^26–31^, there has been a lack of focus on the epidermis as a source of autoantigens initiating the inflammatory loop^20^. To better understand the role of the epidermal microenvironment in the development of autoimmune skin inflammation, we conducted single-cell RNA sequencing (scRNA-seq) of the epidermis of paired lesional skin and non-lesional skin from three psoriasis patients and one healthy donor to elucidate the involved pathogenic immune cell clusters (Supplementary Fig. S1a, b). After unsupervised clustering of > 60,000 cells, we identified four major cell types by their respective signature genes (Supplementary Fig. S1c–e). Strikingly, lymphocyte was exclusively derived from lesional skin (Supplementary Fig. S1f, g, h). To examine cellular heterogeneity of lymphocytes in psoriatic epidermis, we sub-clustered lymphocytes and annotated six subpopulations: CD8^+^ T_RM17_ (cluster 0; *CD8A, ITGAE, IL17A*), CD4^+^ T_reg_ (cluster 1; *CD4, IL2RA, FOXP3*), CD4^+^ T_RM17_ (cluster 2; *CD4, CD69, RORC*), γδT (cluster 3; *TRDC*), CD8^+^ T_cyto_ (cluster 4; *CD8A, GZMK, NKG7*), Cycling CD8^+^ T_RM17_ (cluster 5; *CD8A, ITGAE, IL26, MKI67*) (Fig. 1a–c). Next, we inspected the co-expression of *ITGAE* with *IL17A* and *IL26* (Fig. 1d), demonstrating the existence of T_RM17_ cells in psoriatic epidermis, and found a positive correlation between IL17A-expressing cells and CD8A-expressing cells, yet few CD4^+^ IL17A^+^ cells were observed (Fig. 1e). Surprisingly, we found that CD8^+^ T_RM17_ instead of CD4^+^ T_RM17_ cells, historically identified as the primary source of IL17-producing cells^11^, exhibit the highest T_RM17_ signature and inflammatory cytokine secretion score but relative lower cytotoxicity score (Fig. 1f). We examined the expression of cytokines previously reported to be involved in psoriasis, and found that *IL17A, IL26*, and *CXCL13* were specifically expressed by CD8^+^ T_RM17_ and Cycling CD8^+^ T_RM17_ (Fig. 1g). Notably, most CD8^+^ T_RM17_ and Cycling CD8^+^ T_RM17_ cells were derived from lesional psoriatic skin (Fig. 1h, i), representing proliferation and expansion of those cells within the epidermis. In contrast, CD4^+^ T_RM17_ cells did not show expanded proportion in lesional epidermis. The contribution of CD8^+^ T cells to epidermal inflammation was further confirmed in two mouse strains through depletion of CD4^+^ or CD8^+^ T cells in vivo to explore the indispensable function of CD8^+^ T in driving the epidermal inflammatory phenotype in psoriasis (Supplementary Fig. S2a–j). These data highlight the significance of epidermal CD8^+^ T_RM_ involvement in psoriasis. Considering the role of CD8^+^ T_RM_ cells in relapse of psoriatic inflammation^7^, the mechanisms by which CD8^+^ T_RM_ cells get activated are of great importance.

### *LZTR1*^*high*^ keratinocytes (KCs) have a tight interaction network with CD8^+^ T_RM17_ in psoriatic epidermis

Pathway analysis of differentially expressed genes (DEGs) in Langerhans cells identified multiple pro-inflammatory signaling pathways (Supplementary Fig. S1i, j). Importantly, we identified a distinct KC cluster, Cluster 9, in lesional skin, based on its cluster-identifying genes. This cluster not only exhibited enrichment of well-established psoriasis risk genes, such as *SERPINB4, IL36G*, and *CARD14*^14,32^, but also included a previously less recognized gene, *LZTR1*. Notably, high expression levels of *LZTR1* were associated with increased expression of chemokines, including *CXCL1, CXCL8*, and *CCL20*, within Cluster 9 (Supplementary Fig. S1k, l). Kyoto Encyclopedia of Genes and Genomes (KEGG), and gene set enrichment analysis (GSEA) of DEGs, demonstrated enrichment of psoriasis-related pathways, such as the NOD-like receptor signaling, IL-17 signaling, and NF-κB signaling (Supplementary Fig. S1m, n). Because CD8^+^ T_RM_ cells may have a role in recognizing self-epitopes presented by the binding pockets of HLA-Cw6 or other HLA class I molecules on KC surfaces^33^, we investigated whether *LZTR1*^*high*^ KC exhibits active interaction network with CD8^+^ T_RM17_ cells. To assess this, we analyzed ligand-receptor pairs among *LZTR1*^*high*^ KC and lymphocytes and identified notable interactions in psoriatic epidermis among three cell types: KCs, CD8^+^ T_RM17_, and cycling CD8^+^ T_RM17_, instead of CD4^+^ T_RM17_ (Fig. 1j). Remarkably, the predicted interaction of IL17A and IL17F from CD8^+^ T_RM17_ cells to IL17RA in *LZTR1*^*high*^ KCs stands out as the dominant signaling interaction among lymphocytes (Fig. 1k). To gain the localization information of *LZTR1*^*high*^ KCs with CD8^+^ T_RM17_, we also mapped the expression of *LZTR1*, *CD8A*, and *IL17A* on published spatial-RNA sequencing samples^34^. We reasoned that adjacency in spatial location of *LZTR1*^*high*^ KCs and CD8^+^IL17^+^ T cells would facilitate cell–cell communication (Fig. 1l, m; Supplementary Fig. S1o). Indeed, IL17A-producing CD8^+^ T cells were present in inflamed psoriatic epidermis (Fig. 1n). Thus, the interaction between *LZTR1*^*high*^ KCs and CD8^+^ T_RM17_ presents a novel venue for understanding the regulation of CD8^+^ T_RM17_ cells in psoriatic epidermis.

### LZTR1 deficiency in KCs alleviates skin inflammation

To further support that *LZTR1*^*high*^ KCs represent a distinct subset of epidermal KCs, we analyzed additional scRNA-seq data and observed a significant increase in *LZTR1* expression in psoriatic KCs (Supplementary Fig. S3a), which was further validated in two published bulk RNA-seq datasets by comparing *LZTR1* mRNA expression between lesional and non-lesional skin samples (Supplementary Fig. S3d). Likewise, we observed that *LZTR1*^*high*^ KCs (cluster 11) had prominent expression of inflammatory genes including *CXCL1* and *CXCL2* (Supplementary Fig. S3b, c). To determine the relationship between LZTR1 and inflammatory KC subset in psoriasis, we performed immunofluorescence (IF) staining on skin sections from 10 healthy donors and 20 patients with psoriasis. The staining demonstrated ubiquitous expression of LZTR1 in inflamed psoriatic epidermis compared to healthy skin (Supplementary Fig. S3e, f). In addition, a positive correlation between disease severity in psoriasis (as measured by the Psoriasis Area and Severity Index (PASI)) and LZTR1 protein expression was observed (Supplementary Fig. S3g). This relationship between LZTR1 and skin inflammation was further validated in the imiquimod (IMQ)-induced psoriasis-like mouse model (Supplementary Fig. S3h, i).

**Fig. 2:**
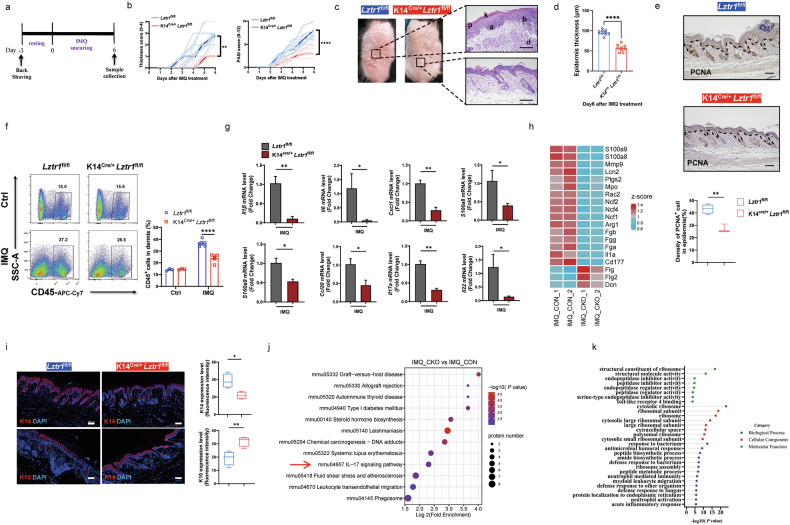
LZTR1 deficiency in KCs alleviates skin inflammation. **a** Experimental schedule for the IMQ model. 62.5 mg cream containing 5% IMQ was applied daily for 6 days on the mouse’s back. **b** Skin thickness score (left) and total PASI score (right) of control (*Lztr1*^fl/fl^) (*n* = 10) and *Lztr1*-deficient (K14^Cre/+^*Lztr1*^fl/fl^) mice (*n* = 9). **c** Macroscopic views (left) and H&E staining (right) on the back from control and *Lztr1*-deficient mice after IMQ application. a acanthosis, b basal layer hyperproliferative KC, d dermal immune cells infiltration, k hyperkeratosis, p parakeratosis. Scale bars, 100 μm. **d** Epidermal thickness quantitation. **e** IHC images of back skin lesions stained with PCNA and corresponding quantitation (*n* = 3–4). **f** FCM analysis of CD45^+^ cell percentage (left) and quantitation (right) in dermis from back skin (*n* = 3–5). **g** qPCR analysis of mRNA encoding chemokines and antimicrobial peptides and cytokines among total mRNA in the back skins (*n* = 3–5). **h** Heatmap of selected gene names based on proteomic data of control (IMQ_CON) and *Lztr1*-deficient (IMQ_CKO) (*n* = 2). **i** IF images of back skin lesions stained with K14, K10 and corresponding quantitation (*n* = 3–4). Scale bars, 100 μm. **j** KEGG pathway enrichment of downregulated proteins (FC < –1.3, *P* < 0.05) from proteomics in *Lztr1*-deficient skin relative to control skin. Log2(Fold Enrichment) on the *x* axis indicates pathway enrichment value among downregulated proteins. **k** GO enrichment of downregulated proteins from proteomics in *Lztr1*-deficient skin relative to control skin (|FC | > 1.3, *P* < 0.05). *P < 0.05, **P < 0.01, ****P < 0.0001 by two-tailed unpaired *t*-test (**d**–**g**, **i**), and two-way ANOVA (**b**). Data are shown as mean ± SEM.

To investigate the definite function of LZTR1 in skin inflammation, we generated epidermis-specific *Lztr1* knockout (KO) mice on C57BL/6 genetic background by crossing mice with loxP-flanked *Lztr1* alleles (*Lztr1*^fl/fl^) with Keratin 14-Cre (K14^Cre/+^) mice (Supplementary Fig. S3j–m). *Lztr1*-deficient (K14^Cre/+^*Lztr1*^fl/fl^) mice exhibited no apparent epithelial tissues defect (Supplementary Fig. 3n, o). To assess the role of LZTR1 in psoriasis pathogenesis, we applied topical IMQ treatment for 6 consecutive days to the back skin of mice (Fig. 2a). Notably, *Lztr1*-deficient mice had a delayed and decreased inflammatory response in the skin compared to *Lztr1*^fl/fl^ controls, without substantial changes in the spleen index or lymph node size (Fig. 2b; Supplementary Fig. S4a–c). Histological examination of the skin lesions in *Lztr1*-deficient mice showed diminished hyperkeratosis and parakeratosis, decreased epidermis thickness (Fig. 2c, d), and a decrease in the number of proliferative KCs in the basal layer (Fig. 2e). We further observed that the skin tissue of *Lztr1*-deficient mice had decreased infiltration of CD45^+^ immune cells (Fig. 2f) as well as suppressed mRNA expression of proinflammatory genes (Fig. 2g). Through proteomic profiling, we identified 79 differentially expressed proteins (|fold change (FC)| > 1.3, *P* < 0.05) in lesional skin between *Lztr1*-deficient and control (*Lztr1*^fl/fl^) mice, including decreased expression of several hallmark psoriasis genes, such as *S100a8*, *S100a9*, *Lcn2*, and *Il1a*, in *Lztr1*-deficient mice (Fig. 2h), along with normalization of epidermal keratin and differentiation markers (keratin 14 (K14), keratin 10 (K10) and filaggrin (Flg)) (Fig. 2h, i). These findings align with the suppression of the IL-17 signaling pathway and other inflammatory responses, as indicated by utilizing KEGG and gene ontology (GO) databases (Fig. 2j, k). In summary, these results demonstrate that intrinsic expression of LZTR1 promotes epidermal inflammation.

### LZTR1 affects IL17A^+^ αβT-cell and KC responses in inflamed skin

**Fig. 3:**
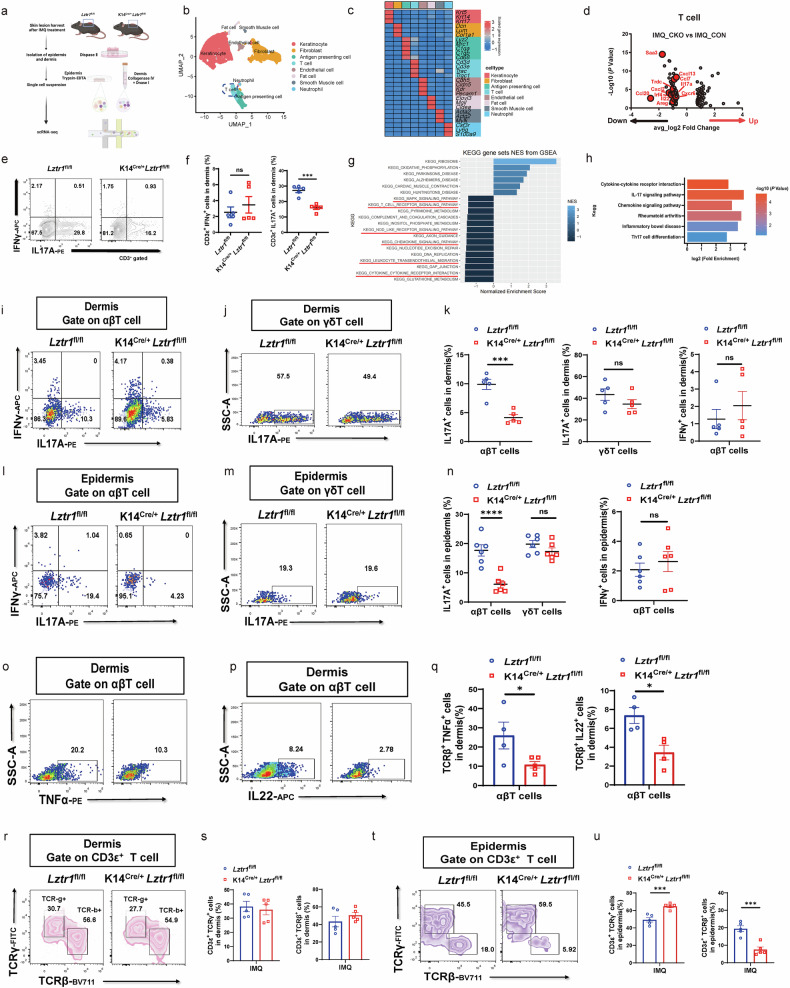
LZTR1 impacts IL17A^+^ αβT cells in inflamed skin. **a** Overview of the workflow of isolating epidermal and dermal cells for scRNA-seq from CKO (*Lztr1*-deficient) and CON (control) mouse lesional skin for scRNA-seq analysis. **b** UMAP of cells in skin lesions of mice. **c** Heatmap displaying relative marker gene expression level among identified cell populations. **d** Volcano plot comparing gene expression of T cells from CKO and CON mice after psoriasis model. **e**, **f** Frequencies of IL17A^+^ or IFNγ^+^ cells among CD3e^+^ cells in the skin (**e**) and summary plot (**f**) (*n* = 5). **g** GSEA analysis of KEGG enrichment on CKO T cells. **h** KEGG enrichment on downregulated DEGs in CKO T cells. Downregulated DEGs were identified as log_2_FC < –1 & *P* < 0.05. Log2(Fold Enrichment) on the *x* axis indicates pathway enrichment value among downregulated genes. **i**–**k** FCM analysis and quantification on IL-17A and IFNγ expression in αβT cells (**i**) and γδT cells (**j**) in dermis and statistic results (**k**) (*n* = 5). **l**–**n** FCM analysis and quantification on IL-17A and IFNγ expression in αβT cells (**l**) and γδT cells (**m**) in epidermis and statistic results (**n**) (*n* = 6). **o**–**q** FCM analysis and quantification on TNF-α (**o**) and IL-22 (**p**) expression in dermal αβT cells and statistic results (**q**) (*n* = 4). **r**, **s** FCM analysis and quantification on the constitution of CD3^+^ T into αβT cells and γδT cells in dermis (**r**) and quantification (**s**) (*n* = 5). **t**, **u** FCM analysis and quantification on the constitution of CD3^+^ T into αβT cells and γδT cells in epidermis (**t**) and quantification (**u**) (*n* = 5). ns: not significant; *P < 0.05, ***P < 0.001, ****P < 0.0001 by two-tailed unpaired *t*-test (**f**, **k**, **n**, **q**, **s**, **u**). Data are shown as mean ± SEM.

To determine the effect of epidermal *Lztr1* KO on immune cell function and cellular responses, we used scRNA-seq to analyze all cell types present in mouse skin at the experimental endpoint (Fig. 3a–c). Given the suppressed inflammatory phenotype in *Lztr1*-deficient mice, we prioritized our analyses on immune cell subsets. Intriguingly, genes involved in T17 (Th17, Tc17) differentiation, such as *Il17a, Il22, Irf4*, and *Cxcr6*, were decreased in *Lztr1*-deficient mice (Fig. 3d), further validated by flow cytometry (FCM) (Fig. 3e, f). Notably, one of the most pronounced changes in T cells, derived from *Lztr1*-deficient mouse skin, were decreased signatures of genes involved in T-cell receptor signaling and Th17 cell differentiation (Fig. 3g, h). In contrast, we did not observe activation changes in other immune cell subsets using GSEA (Supplementary Fig. S5a–d), nor did we observe differences in the frequencies of CD11b^+^F4/80^+^ macrophage, CD11c^+^I-A/I-E^+^ dendritic cell (DC), or CD11b^+^Ly6G^+^ neutrophils by FCM (Supplementary Fig. S5f–h). These findings suggest that LZTR1 deficiency leads to a failure of differentiation and activation of T cells in inflamed skin.

To determine what drives the variation in the IL17A secretion capacity of T cells in the IMQ-induced inflammatory model, we separated T cells into αβT and γδT subsets. We found that the percentage of infiltrating IL17A^+^ αβT was diminished in both the epidermis and dermis of *Lztr1*-deficient mice, whereas the ratio of IL17A^+^ γδT remained the same (Fig. 3i–n). Moreover, other psoriasis-related pathogenic cytokines like IL22 and TNF-α were downregulated in αβT from *Lztr1*-deficient mice (Fig. 3o–q). Notably, even though the percentage of CD3ε^+^ T cells was not altered after *Lztr1* gene deletion (Supplementary Fig. S5e), there was a clear decline in the frequency of αβT cells in the epidermis but not in the dermis (Fig. 3r–u). Therefore, the major effect of *Lztr1* gene deletion was diminished differentiation and infiltration of IL17A^+^ αβT cells in inflamed epidermis.

To determine the impact of LZTR1 deficiency in KCs, we focused the scRNA-seq analyses on KCs. Further examination of gene signature in KCs indicated decreased expression of various psoriasis hallmark genes, such as *S100a8, S100a9* and *Cxcl1* in K14^Cre/+^*Lztr1*^fl/fl^ KCs compared to *Lztr1*^fl/fl^ KCs (Supplementary Fig. S6a). Pathway enrichment analysis discovered downregulated genes involved in multiple inflammatory pathways, including IL-17 signaling pathway and TNF signaling pathway (Supplementary Fig. S6b, c). We also observed a marked difference in KCs distribution on the uniform manifold approximation and projection (UMAP) between *Lztr1*-deficient and control IMQ-treated mice (Supplementary Fig. S6d), representing a shift in transcriptional signature and cell proportions, especially for Cluster 2 and Cluster 6, which were nearly absent in K14^Cre/+^*Lztr1*^fl/fl^ mice (Supplementary Fig. S6e). Notably, these two clusters expressed markers of inflammatory response, similar to psoriasis KCs, reflective of acanthosis, inflammatory infiltration, parakeratosis, and antigen presentation (Supplementary Fig. S6f–j)^14,35^. Notably, the expression of autoantigens, including *Camp* (LL-37), *Krt6a*, *Krt6b*, and *Krt16*, sharing homology with M-protein^18,36^, was consistently decreased in *Lztr1*-deficient mice (Supplementary Fig. S6k). This suggests a reduced potential to activate CD8^+^ T_RM17_ cells in the absence of LZTR1. Specifically, KCs from *Lztr1*-deficient mice showed a well-distributed differentiation trajectory on pseudotime, while in comparison with control mice, there was a shift towards a more active pro-inflammatory and abnormally differentiated state (Supplementary Fig. 6l–o). We clustered genes identified as significantly covarying with pseudotime and defined groups of genes through GO enrichment along the pseudotime (Supplementary Fig. S6p). Given the visible discrepancy between *Lztr1*-deficient and control KCs, we first determined whether LZTR1 directly affects KC function and differentiation. However, we failed to observe any effects of LZTR1 on cell proliferation and inflammation in either HaCaT cells (KC cell line) or primary KCs after *LZTR1* KO (Supplementary Fig. S7a–l).

Therefore, we speculate that the ameliorated inflammatory response observed in the absence of LZTR1 may be attributed to changes in the immune environment rather than intrinsic regulation of inflammatory function in KCs. Indeed, according to the cell-chat analysis, we concluded that the protective effect of *Lztr1* gene deletion in KCs on epidermal inflammation was secondary to a reduction in interaction with immune cells (Supplementary Fig. S7m–o), particularly decreased αβT cell-KC interactions on pro-inflammation signals such as CXCL and CCL (Supplementary Fig. S7p–r).

### The attenuated skin inflammatory response observed in *Lztr1*-deficient mice is CD8^+^ T cell dependent

The T cell number we captured in our initial scRNA-seq data of mouse skin was insufficient for deeper analysis of involved T cell subsets (data not shown). To address this, we performed scRNA-seq on IMQ-treated skin after sorting CD45^+^ cells to enrich for immune cells (Supplementary Fig. S8a–c, e). Using this larger dataset of immune cells, we identified two clusters with enriched T17 differentiation (Supplementary Fig. S8f), named CD8^+^ Tc17-like and T_RM_, both with reduced frequency in *Lztr1*-deficient mice (Supplementary Fig. S8d).

**Fig. 4:**
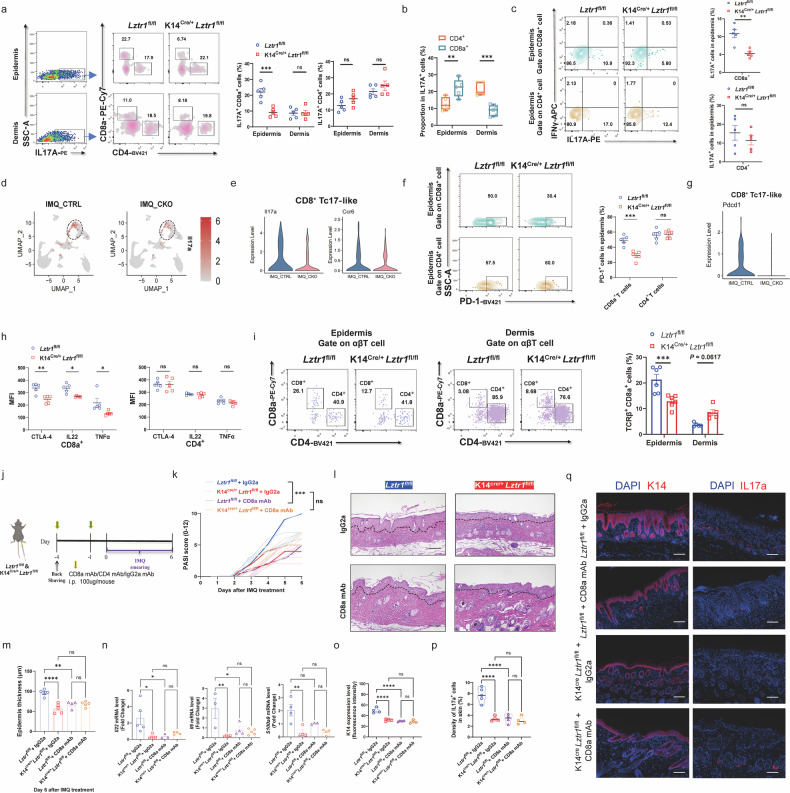
The attenuated skin inflammatory response observed in *Lztr1*-deficient mice is CD8^+^ T cell dependent. **a** FCM analysis of IL17A^+^ cells in dermis and epidermis, and the percentage of CD4^+^ T and CD8^+^ T among IL17A^+^ live cells (left), and quantification (right) (*n* = 5). **b** Proportion of CD4^+^ T and CD8^+^ T among IL17A^+^ live cells in WT mice of psoriasis model (*n* = 5). **c** Frequencies of IL17A^+^ or IFNγ^+^ cells among CD4^+^ T and CD8^+^ T in inflamed IMQ-treated mouse epidermis (left), and quantification of IL17A^+^ cells (right) (*n* = 5). **d** Feature plot of *Il17a* mRNA expression derived from scRNA-seq data after sorting with CD45^+^. **e** Violin plots of *Il17a* and *Ccr6* expression in CD8^+^ Tc17-like cells. **f** FCM and quantifications of PD-1^+^ cells in CD8a^+^ T cells and CD4^+^ T cells from epidermis (*n* = 5). **g** Violin plot presenting the expression level of *Pdcd1* in CD8^+^ Tc17-like cells from CTRL and CKO mice. **h** Mean fluorescence intensity (MFI) of CTLA-4, IL22, TNF-α expression in epidermal CD4^+^ T and CD8^+^ T from inflamed IMQ-treated mice (*n* = 5). **i** FCM of CD8a^+^ and CD4^+^ among αβT cells in epidermis and dermis and quantification of TCRβ^+^ CD8a^+^ cells proportion (*n* = 5–6). **j** Workflow for CD8^+^ T depletion and psoriasis induction. **k**–**m** PASI score (**k**), H&E staining (**l**), and epidermal thickness (**m**) of dorsal lesions. Scale bars, 100 μm (*n* = 4–5). **n** qPCR of mRNA encoding cytokines and antimicrobial peptides in mice skin (*n* = 3–5). **o**–**q** IF assay quantitation by K14 fluorescence intensity (**o**) and IL17A^+^ fluorescent dots percentage (**p**) in mouse lesional skin with images of K14 and IL17A (**q**) expression in back skin lesions (*n* = 4–5). Scale bars, 100 μm. ns: not significant; *P < 0.05, **P < 0.01, ***P < 0.001, ****P < 0.0001 by two-tailed unpaired *t*-test (**c**, **h**), one-way ANOVA (**m**–**p**) and two-way ANOVA (**a**, **b**, **f**, **i**, **k**). Data are shown as mean ± SEM.

We categorized IL17A^+^ αβT cells into CD4^+^ or CD8^+^ T cells to identify which cell type exhibits a defect in IL17A secretion in the epidermis and dermis. *Lztr1*-deficient mice exhibited a decreased proportion of CD8^+^ T cells among IL17A^+^ lymphocytes exclusively in the epidermis, while the CD4^+^ T cell population remained unaffected in both the epidermis and dermis (Fig. 4a). The pathogenic role of CD8^+^ T cells in psoriasis, characterized by the release of cytokines such as IL17A and IL22, has been extensively elucidated^34,37^. In the present study, we showed that the majority of IL17A^+^ T cells in the psoriatic epidermis were CD8^+^ T cells, whereas IL17A^+^CD4^+^ T cells were more frequent in the dermis (Fig. 4b), in line with our scRNA-seq results in psoriatic epidermis (Fig. 1f, g). Remarkably, our data demonstrated a specific impairment in the differentiation of Tc17 cells within the epidermis of *Lztr1*-deficient mice (Fig. 4c–e), accompanied by decreased expression of T activation-related proteins in psoriasis, such as CTLA-4, PD-1, IL22 and TNF-α (Fig. 4f–h)^12,38,39^. In stark contrast, expression of these activation makers, including IL17A, PD-1, CTLA-4, IL22, and TNFα, was similar in CD4^+^ T cells between *Lztr1*-deficient and control mice (Fig. 4c, f, h). Furthermore, *Lztr1*-deficient mice had fewer CD8^+^ T cells in the epidermis but with a slight, potential compensatory, increase in the dermis (Fig. 4i), implying failed entry of CD8^+^ T into the epidermis. Together, these data demonstrate that LZTR1 supports epidermal CD8^+^ T activation and migration.

To determine whether systemic immune responses are affected in *Lztr1*-deficient mice, we analyzed CD4^+^ and CD8^+^ T cells from the spleen, inguinal lymph nodes (iLNs), and peripheral blood mononuclear cells (PBMCs) from IMQ-treated mice and observed no alteration in cell frequency or production of interferon-gamma (IFNγ) or IL17A (Supplementary Fig. S9a–c), suggesting no effect on global T-cell responses.

We demonstrated that depletion of CD8^+^ T cells ameliorates the disease severity in control mice, while abolishing CD8^+^ T in *Lztr1*-deficient mice had no additive impact on disease severity (as measured by PASI) (Fig. 4j, k), epidermal thickness (Fig. 4l, m), inflammatory response (Fig. 4n, p, q), or hyperkeratosis (Fig. 4o, q). In contrast, depletion of CD4^+^ T cells had an additive anti-inflammatory effect on *Lztr1*-deficient mice (data not shown), and a substantial psoriatic phenotype difference remained between *Lztr1*-deficient and control mice after application of IMQ (Supplementary Fig. S9d–g). Moreover, anti-CD4 treated *Lztr1*-deficient mice had reduced mRNA expression of *Il1β, Il17a, Cxcl1* and *S100a9* in lesion (Supplementary Fig. S9h), limited IL17A secretion in T cells (Supplementary Fig. S9i, j), and fewer IL17A, CTLA-4 and PD-1 protein expression in CD8^+^ T cells (Supplementary Fig. S9k, l), compared with anti-CD4-treated control mice. The phenotype difference observed after depleting either CD4^+^ or CD8^+^ T cells in *Lztr1*-deficient mice emphasizes that the attenuation of the inflammatory epidermal response in *Lztr1*-deficient mice is CD8^+^ T dependent.

### *Lztr1*-deficient KCs fail to activate CD8^+^ T_RM_ during immune rechallenge

After elucidating the regulation of epidermal Tc17 by LZTR1, we refocused our attention on our initial discovery that *LZTR1*^*high*^ KCs engage in an undiscovered crosstalk with CD8^+^ T_RM17_ through IL-17 signaling. Building upon previous evidence suggesting that epidermal LZTR1 can modulate Tc17 function, we hypothesized that LZTR1 may also regulate the activation of T_RM17_. CD8^+^ T_RM_ cells are found in barrier tissues, including skin, intestine, lung, and genitourinary tract, allowing rapid response to pathogenic stimuli^40^.

**Fig. 5:**
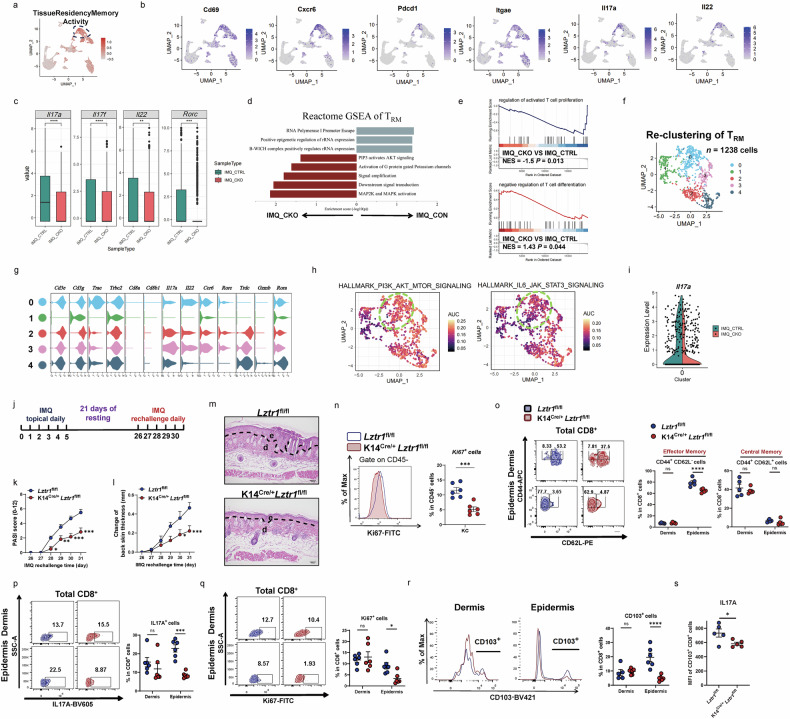
*Lztr1*-deficient KCs fail to activate CD8^+^ T_RM_ during immune rechallenge. **a** Feature plot of T_RM_ activity score overlayed on UMAP distribution of T_RM_ cells. **b** Feature plot of T_RM_ feature genes. **c** Bar chart of the expression of T17 differentiation genes in T_RM_ cells. **d** Bar chart showing GSEA analysis of T_RM_. **e** Enriched GO pathway of T_RM_ by GSEA analysis. **f** UMAP plot of 5 T_RM_ subclusters. **g** Violin plot showing the representative genes expressed in 5 clusters. **h** AUC score of hallmark pathway with UMAP distributions in T_RM_. **i** Violin plot illustrating *Il17a* expression level in Cluster 0 (CD8^+^ T_RM_). **j** Overview of the workflow for immune rechallenge. **k**, **l** Line graph representing the changes in PASI (**k**) and back skin thickness (**l**) (*n* = 6). **m** H&E images. Scale bars, 100 μm. **n** FCM and quantification of Ki67 expression in CD45^*−*^ cells (KC) (*n* = 6). **o**–**r** FCM and quantification on percentage of CD44^+^CD62L^*−*^ and CD44^+^CD62L^+^ expression in CD8^+^ T cells (**o**) and percentage of IL-17A^+^ (**p**), and Ki67^+^ (**q**) in CD8^+^ T cells. Respective statistic plots on the right (*n* = 5–6). **r** FCM univariate histogram and quantification of CD103^+^CD8^+^ T_RM_ cells in dermis and epidermis (*n* = 5–6). **s** MFI of IL-17A released by CD103^+^CD8^+^ T_RM_ cells in epidermis (*n* = 5). ns: not significant; *P < 0.05, **P < 0.01, ***P < 0.001, ****P < 0.0001 by Wilcoxon rank test (**c**), two-tailed unpaired *t*-test (**n**, **s**) and two-way ANOVA (**k**, **l**, **o**–**r**). Data are shown as mean ± SEM.

To investigate CD8^+^ T_RM_ cells in inflamed psoriatic epidermis, we analyzed the T_RM_ (Cluster 3) identified in our scRNA-seq data. T_RM_ cells highly expressed transcripts related to tissue residency memory, such as *Cd69, Itgae, Cxcr6*, and *Il17a* (Fig. 5a, b**;** Supplementary Fig. S8b)^41^. Gene expression profile of T_RM_ showed lower expression of T17 genes (*Il17a, Il17f, Il22*, and *Rorc*) and suppressed expression of genes involved in T cell differentiation and proliferation in *Lztr1*-deficient mice compared to control mice after IMQ application (Fig. 5c–e). Notably, among the downregulated pathways in the IMQ_CKO group, the PI3K-AKT pathway stands out as closely associated with the survival and formation of T_RM_^42^, suggesting that the survival of T_RM_ may be impaired in *Lztr1*-deficient mice (Fig. 5d). Among the T_RM_ (Fig. 5f), Cluster 0 represented CD8^+^ T_RM_, characterized by the expression of *Trac*, *Cd8a*, *Cd8b1*, *Il17a*, *Il22*, and demonstrating high area under the curve (AUC) score for T17 cell relevant differentiation pathways (Fig. 5g, h). CD8^+^ T_RM_ in *Lztr1*-deficient mice had lower expression levels of *Il17a* (Fig. 5i).

To determine the relevance of intraepidermal CD8^+^ T_RM_ during immune rechallenge, we used the IMQ model to mimic psoriasis recurrence (Fig. 5j)^11,43^, enabling us to observe the response of memory T cells. As expected, skin thickness and disease severity (modified PASI) were lower in *Lztr1*-deficient mice upon re-exposure to topical IMQ (Fig. 5k, l), which was further confirmed by skin histology and FCM for Ki67 in KCs (Fig. 5m, n).

Given the marked differences in epithelial responses and inflammation on rechallenge between *Lztr1*-deficient and control mice, we wanted to address the role of CD8^+^ T_RM_ cells in this process. To do this, we assessed the expression of the effector memory marker CD44 and the naïve marker CD62L by dividing T lymphocytes into CD44^+^CD62L^*−*^ (effector memory), CD44^+^CD62L^+^ (central memory), and CD44^*−*^CD62L^+^ (naïve)^44^. We observed a decreased frequency of CD8^+^ CD44^+^CD62L^*−*^ cells in the epidermis rather than the dermis of *Lztr1*-deficient mice with FCM (Fig. 5o). Consistent with this, decreased IL17A expression and proliferation rate were observed in CD8^+^ T upon IMQ rechallenge (Fig. 5p, q). Meanwhile, the proportion of CD103^+^CD8^+^ T cells (CD8^+^ T_RM_) and the production of IL17A from CD8^+^ T_RM_ were consistently decreased in the epidermis of *Lztr1*-deficient mice (Fig. 5r, s). Therefore, our findings underscore the significance of LZTR1 in the epidermis for facilitating the activation and proliferation of CD8^+^ T_RM_ during immune rechallenge, which validates the regulatory role of *LZTR1*^*high*^ KCs in modulating the inflammatory response of CD8^+^ T_RM17_.

### LZTR1 modulates the function of CD8^+^ T cells by regulating the expression of MHC-I in KCs

**Fig. 6:**
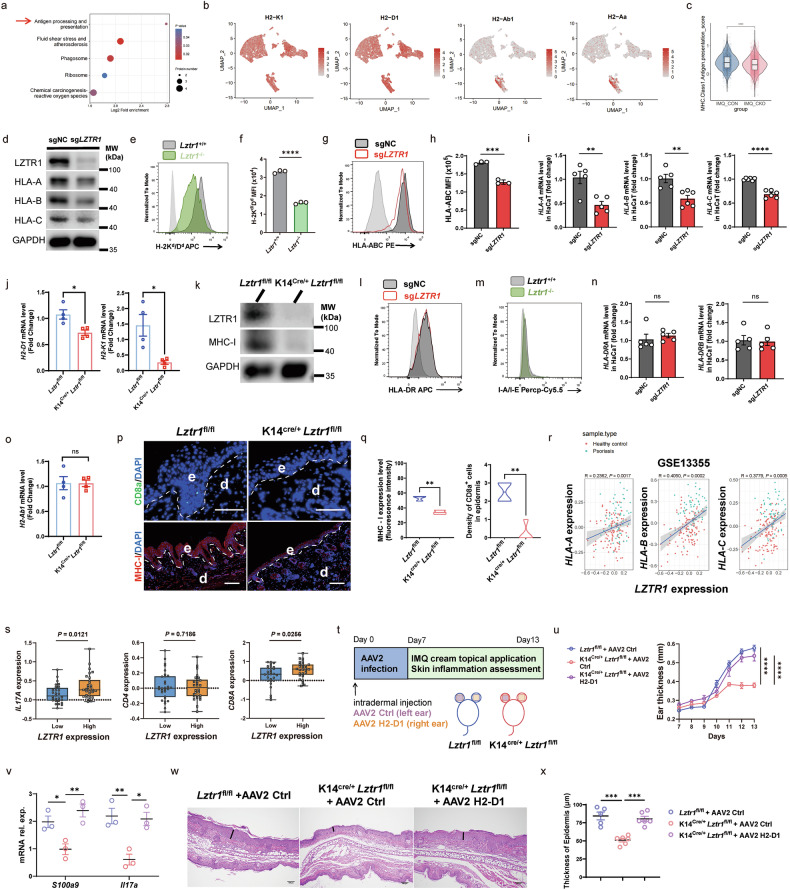
LZTR1 modulates the function of CD8^+^ T cells by regulating the expression of MHC-I in KCs. **a** Dot plot of KEGG pathway enrichment on protein with downregulated phospho-site (FC <–1.3, *P* < 0.05) in *Lztr1*-deficient mice. Log2 Fold enrichment on the *x* axis indicates pathway enrichment value among downregulated proteins. **b** Feature plot of gene expression on total cells from mouse skin tissue. **c** MHC-I antigen presentation score on KCs. **d**–**h** Analysis of MHC-I molecular expression in HaCaT transfected with *sgLZTR1* or CTRL sgRNA or primary KCs extracted from *Lztr1*-deficient or Control mice. IB (**d**) and FCM analysis (**e**, **g**) of MHC-I molecular membrane expression and quantification (**f**, **h**) (*n* = 3). **i**, **j** qPCR of mRNA encoding MHC-I molecular genes among HaCaT cell (**i**) (*n* = 5–6) and mice skin lesions (**j**) (*n* = 4). **k** IB analysis of MHC-I molecules in mouse epidermis tissue. **l**, **m** FCM of MHC-II molecular membrane expression in HaCaT (**l**) or primary mouse KCs (**m**). **n**, **o** qPCR analysis of MHC-II molecular genes among HaCaT (**n**) (*n* = 5) and mouse skin lesions (**o**) (*n* = 4). **p**, **q** IF images (**p**) and quantification (**q**) of CD8a number per sight (×200) and MHC-I expression (×100) in skin lesional sections. Scale bars, 100 μm (*n* = 3–4). **r** Linear regression analysis of *LZTR1* and *HLA-A*, *HLA-B*, *HLA-C* mRNA expression among psoriasis lesion and healthy control based on GSE13355. **s** Box plot showing mRNA expression levels of *IL17A*, *CD4*, and *CD8A* in samples from patients with psoriasis divided into “*LZTR1*^low^” group (low half of expression) and “*LZTR1*^high^” group (top half of expression) based on GSE13355. **t** Experimental schedule. Each mouse was intracutaneously injected with AAV2-Ctrl or AAV2-H2-D1 on each ear and then treated with 15 mg cream containing 5% IMQ onto each ear daily for another 6 days. **u** Ear thickness change (*n* = 5–6). **v** qPCR of mRNA encoding *S100a9*, *Il17a* in mouse skin lesions (*n* = 3). **w**, **x** H&E of ear lesion skin (**w**) and summary (**x**). Scale bars, 100 μm (*n* = 5–6). ns: not significant; *P < 0.05, **P < 0.01, ***P < 0.001, ****P < 0.0001 by Wilcoxon rank test (**c**), two-tailed unpaired *t*-test (**f**, **h**–**j**, **n**, **o**, **q**, **s**), one-way ANOVA (**v, x**) and two-way ANOVA (**u**). Data are shown as mean ± SEM.

To explore the mechanism by which LZTR1 in KCs regulates epidermal CD8^+^ T cell activation, we performed proteomic profiling of mouse skin followed by pathway enrichment analysis. This demonstrated that proteins associated the antigen processing and presentation were suppressed in the inflamed skin of *Lztr1*-deficient (K14^Cre/+^*Lztr1*^fl/fl^) mice (Fig. 6a). MHC-I molecules are ubiquitously expressed on the surfaces of nucleated cells (Fig. 6b), encoded by the HLA genes in humans and the H-2 genes in mice^45^. Patients with cutaneous psoriasis exhibit a significant overlap in the genetic variants predisposing them to the conditions, particularly the association with HLA-Cw6. Furthermore, other class I antigens, such as HLA-B13, HLA-B57, HLA-B39, and HLA-Cw7, are also associated with psoriasis^33^. Similar results were noted regarding the heightened expression of MHC-I molecules on lesional KCs in psoriasis (Supplementary Fig. S10a, b).

Similar evidence was provided in the scRNA-seq data, where we observed a lower module score for MHC-I antigen presentation in *Lztr1*-deficient (K14^Cre/+^*Lztr1*^fl/fl^) KCs (Fig. 6c). Consistent with this, we observed loss of both mRNA and protein expression of MHC-I in sg*LZTR1* HaCaT, *Lztr1*^*−/−*^ primary KC, and *Lztr1*-deficient mouse epidermis, along with decreased infiltration of epidermal CD8^+^ T cells in *Lztr1*-deficient mice (Fig. 6d–k, p, q). There were no observed alterations in the expression of MHC class II molecules (Fig. 6l–o). Correlation analysis in human inflamed psoriatic skin, using RNA-seq data from healthy (or non-lesional skin) and lesional psoriatic skin, showed a positive correlation between *LZTR1* mRNA expression and mRNA expression of *HLA-A, HLA-B*, and *HLA-C* (Fig. 6r; Supplementary Fig. S10d–g), consistent with the intensity and co-localization of LZTR1 and HLA-C based on IF staining of lesional psoriatic skin (Supplementary Fig. S10c). Notably, patients with high expression of *LZTR1* (*LZTR1*^*high*^) had higher levels of mRNA expression of *CD8A*, and *IL17A* rather than *CD4* mRNA expression (Fig. 6s), suggesting a causal relationship between *LZTR1*^*high*^ KCs and Tc17 or CD8^+^ T_RM17_ responses.

To investigate whether MHC-I upregulation in KCs promotes inflammation through activation of epidermal CD8^+^ T cells, we injected H2-D1-expressing adeno-associated virus serotype 2 (AAV2 H2-D1) intracutaneously into mouse ear to increase H2-D1 expression in the epidermis followed by topical IMQ treatment (Supplementary Fig. S10h, i). Epidermal thickness and proportion of epidermal, rather than dermal, CD8^+^IL17A^+^ and CD8^+^CD44^+^ cells were notably increased in IMQ-treated mice that received AAV2 H2-D1 compared with mice administered with AAV-Ctrl (Supplementary Fig. S10j–m), suggesting that continuous MHC-I antigen presentation in KCs is crucial for epidermal CD8^+^ T cell differentiation and cytokines release, but it does not affect the function of CD8^+^ T cell in dermis. Furthermore, using an in vitro system, we observed that to achieve full T-cell activation, whether in CD4^+^ or CD8^+^ T cells, continuous anti-CD3 stimulation (first signal) was indispensable (Supplementary Fig. S11a–d), indicating that spatial proximity of MHC-I interactions with intraepidermal CD8^+^ T cells mediates epidermal inflammatory responses.

To determine whether the effect of *Lztr1*-deficiency is mediated by the lack of MHC-I expression, we designed a rescue experiment in *Lztr1*-deficient mice by exogenously expressing MHC-I (Fig. 6t). Consistent with our prior observations, intradermal overexpression of H2-D1 completely restored inflammatory responses in *Lztr1*-deficient mice (Fig. 6u–x). Lastly, to determine if this was dependent upon the function of LZTR1 in KCs, we crossed *Lztr1*^fl/fl^ mice with Lysozyme 2-Cre (Lyz2^Cre/+^) mice to specifically delete *Lztr1* gene in professional antigen-presenting cells but did not find differences in inflammatory responses between *Lztr1*^fl/fl^ mice and Lyz2^Cre/+^*Lztr1*^fl/fl^ mice (Supplementary Fig. S12a–c), suggesting that the antigen presentation function of KCs affects the CD8^+^ T cells in psoriasis lesions. Altogether, given that CD8^+^ T_RM_ cells are reactivated by MHC-I signaling^46^, these data indicate that the increased MHC-I antigen presentation in KCs is a primary cause of abnormal and heightened activation of CD8^+^ T_RM17_, in an LZTR1-dependent manner.

### Depletion of LZTR1 in KCs restricts NF-κB1 p50 binding to HLA class I promoter

**Fig. 7:**
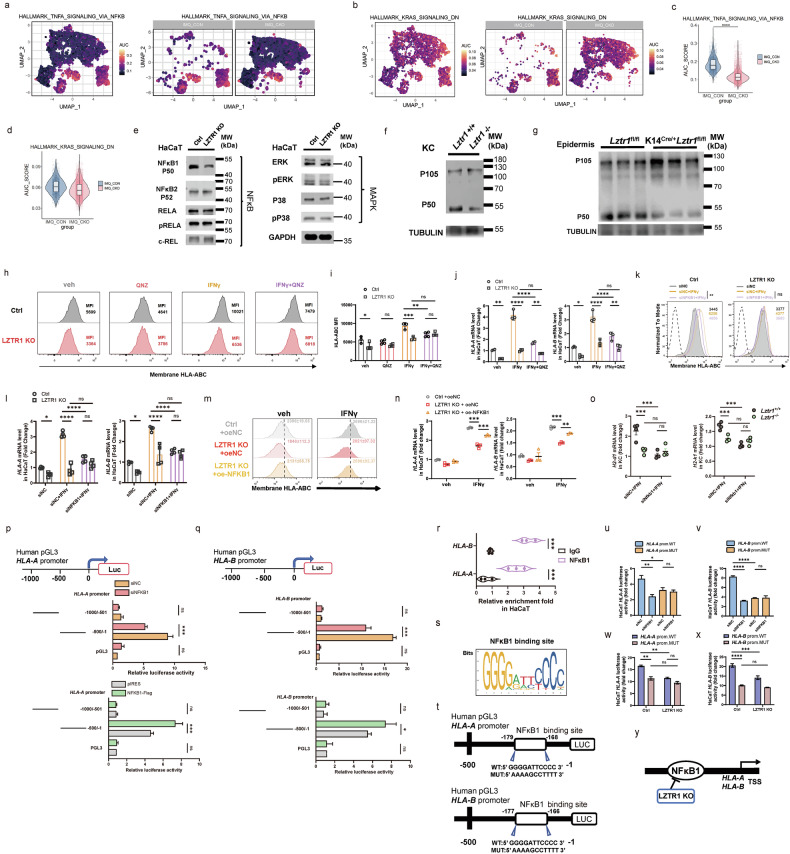
Depletion of LZTR1 in KCs restricts NF-κB1 p50 binding to HLA class I promoter. **a**, **b** Hallmark pathway of TNFA_NFKB (**a**) and KRAS (**b**) signaling AUC score cluster distributions. **c**, **d** Violin plots of the AUC score in TNFA_NFKB (**c**) and KRAS (**d**) signaling in KCs. **e**–**g** IB analysis of the indicated proteins of NF-κB and MAPK signaling pathway in HaCaT after treatment with *LZTR1* sgRNA (**e**), primary mouse KC (**f**), and mouse epidermis (**g**). **h**–**n** MFI level of membrane HLA-ABC or mRNA level of HLA-A, HLA-B in Ctrl and *LZTR1* KO HaCaT treated with 5 μM NF-κB inhibitor QNZ (**h**–**j**), si*NFKB1* (**k**, **l**) or overexpressed (oe)-*NFKB1* (**m**, **n**). veh, vehicle (*n* = 2–4). **o** qPCR of H2-D1 and H2-K1 mRNA expression in KCs from mice treated with si*Nfkb1* and IFNγ (*n* = 4). **p**, **q** Schematic diagram of promoter site construction and results for dual luciferase assay of HLA-A (**p**) and HLA-B (**q**) with si*NFKB1* or oe-*NFKB1* (*n* = 3). **r** Relative enrichment fold of HLA-A and HLA-B promoter sequence immunoprecipitated with NF-κB1 in HaCaT analyzed by qPCR (*n* = 3). **s** NF-κB1 predictive binding site on HLA-A and HLA-B promoter. **t** Schematic diagram of NF-κB1-binding deficiency promoter construction. **u**–**x** Quantification of luciferase activity level of HLA-A and HLA-B in WT or mutant promoter combined with si*NFKB1* (**u**, **v**) and *LZTR1* KO (**w**, **x**) (*n* = 3). **y** Schematic mechanism diagram of LZTR1 regulating MHC-I. ns: not significant; *P < 0.05, **P < 0.01, ***P < 0.001, ****P < 0.0001 by Wilcoxon rank test (**c**), one-way ANOVA (**k**) and two-way ANOVA (**i**, **j**, **l**, **n**–**r**, **u**–**x**). Data are shown as mean ± SEM.

To investigate how LZTR1 regulates MHC-I expression, we focused on two principal signaling pathways, RAS-MAPK and NF-κB, regulated by LZTR1^24,25^. Intriguingly, we found that AUC score of TNF_SIGNALING_VIA_NFKB pathway was inhibited in *Lztr1*-deficient mouse KCs (Fig. 7a–d). These data were supported by a decrease in NF-κB1 (p50) levels after treatment with *LZTR1* sgRNA and further validated in *Lztr1*^*−/−*^ KC and *Lztr1*-deficient mouse epidermis (Fig. 7e–g). On the contrary, we did not observe any changes in the levels of MAPK or other NF-κB family members, nor in the NFKB1 mRNA level (Fig. 7e; Supplementary Fig. S10n–p).

NF-κB1 encodes two functionally distinct proteins termed p105 (precursor form) and p50 (active form)^47^, attending the transcriptional process of MHC-I molecules (Supplementary Fig. S10q). To test whether NF-κB signaling underlies the downregulation of MHC-I in the absence of LZTR1, we treated *LZTR1*-KO HaCaT or *Lztr1*^*−/−*^ primary KC with NF-κB inhibitor (QNZ), si*NFKB1* vs overexpression of NFKB1, under IFNγ co-stimulation to induce MHC-I expression, and measured MHC-I expression using several different assays. Indeed, these data demonstrated that LZTR1 deficiency leads to downregulation of MHC-I expression through restraining NF-κB1 activity (Fig. 7h–o).

To investigate whether *HLA-A* and *HLA-B* are direct target genes for NF-κB1, we locked 500 bp upstream from their transcription start sites (TSS) using a dual luciferase reporter assay (Fig. 7p, q) and searched for potential NF-κB1-binding sites in the human *HLA-A* and *HLA-B* gene promoter region (Fig. 7s). This was then confirmed by qPCR detection of *HLA-A* and *HLA-B* promoter sequences immunoprecipitated with NF-κB1 (Fig. 7r). In addition, we generated promoter constructs (–500 ~ –1 bp) containing a single mutation to cause specific NF-κB1-binding deficiency (Fig. 7t). Notably, this approach was successful in the control group but failed to provide an additional level of inhibition after si*NFKB1* or in *LZTR1*-KO KCs (Fig. 7u–x). Therefore, this evidence supports the notion that LZTR1 regulates MHC class I molecules by inhibiting NF-κB signaling, specifically by suppressing the generation of NF-κB1 (p50). NF-κB1 is a transcriptional regulator for *HLA-A* and *HLA-B* by binding to their specific promoter regions. (Fig. 7y).

### Contribution of LZTR1 to CD8^+^ T cell crosstalk in gastrointestinal epithelium in the DSS colitis model of IBD

Given LZTR1’s involvement in controlling the activation of epidermal CD8^+^ T cells, we were intrigued to investigate whether these interactions between CD8^+^ T cells and epithelial cells also play a role in the pathology of IBD^19^. As Krt14 is universally expressed in epithelial tissues^48^, we applied dextran sulfate sodium salt (DSS) to induce colitis in mice to examine the function of LZTR1 on intestinal epithelial cells during colitis. Loss of LZTR1 in epithelium alleviated loss of body weight, decreased disease activity index (DAI), colon shortening, and splenomegaly (Supplementary Fig. S13a–e)^49^. Histological injuries in the proximal colon, including degradation of tissue structures, disruption of the epithelial barrier, and inflammatory cell infiltration, were attenuated in *Lztr1*-deficient mice (Supplementary Fig. S13f, g). This phenotype was accompanied by markedly decreased expression of TNF-α, CD44, and IL17A in intraepithelial CD8^+^ lymphocytes (IELs) without affecting the frequency of CD8^+^ T cells in mesenteric lymph nodes (mLNs) (Supplementary Fig. S13h–l), indicating that similar to its role in the epidermis, LZTR1 in intestinal epithelial cells also regulates the activation of epithelial CD8^+^ T cells. Furthermore, we evaluated the contribution of CD8^+^ T cells to colitis by administering specific anti-CD4 and anti-CD8α depleting antibodies before DSS induction, compared with anti-CD8α treatment, *Lztr1*-depleted mice following anti-CD4 or IgG isotype administration showed alleviated intestinal injuries (Supplementary Fig. S13m–p), providing additional evidence for the interactions between epithelial LZTR1 and CD8^+^ T cell activation in additional autoimmune disease model.

### LZTR1 primes proteasome-ribosome interactions and prompts co-translational biogenesis of NF-κB1 P50

**Fig. 8:**
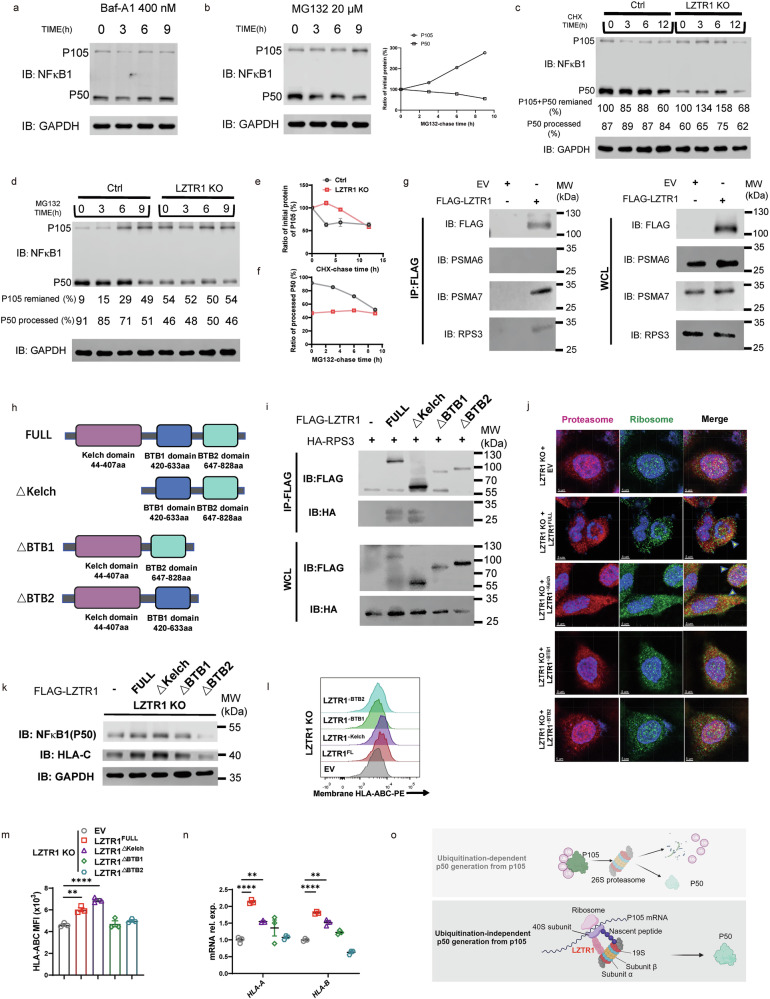
LZTR1 primes proteasome-ribosome interactions and prompts co-translational biogenesis of NF-κB1 P50. **a**, **b** IB analysis of HaCaT treated with 400 nM Baf-A1 (**a**) and 20 μM MG132 (**b**) for the indicated times. Right: summary plot of IB. **c**–**f** Ctrl and *LZTR1* KO HaCaT cells were treated with 50 μg/mL CHX (**c**) and 20 μM MG132 (**d**) for the indicated times, and corresponding NF-κB1 quantification (**e**, **f**). **g** IB analysis of FLAG-LZTR1, PSMA6, PSMA7, and RPS3 in FLAG-LZTR1 precipitates immunoprecipitated from HaCaT cells reconstituted with either empty vector (EV) or FLAG-tagged human LZTR1. WCL: whole cell lysate. **h** Schematic domain structure of LZTR1 and design on domain mutation. **i** IB analysis of FLAG-LZTR1 and HA-RPS3 in FLAG-LZTR1 precipitates immunoprecipitated from HaCaT cells transfected with EV (−), FLAG-LZTR1 (FULL), FLAG-LZTR1^ΔKelch^ (ΔKelch), FLAG-LZTR1^ΔBTB1^ (ΔBTB1), FLAG-LZTR1^ΔBTB2^ (ΔBTB2) along with HA-RPS3. **j** IF of proteasome alpha unit and RPS3 in *LZTR1* KO HaCaT cells transfected with different plasmid by confocal microscopy. Scale bars, 5 μm. **k** IB analysis of NF-κB1 (p50) and HLA-C expression in *LZTR1* KO HaCaT cells additionally transfected with EV (−), FLAG-LZTR1 (FULL), FLAG-LZTR1^ΔKelch^ (ΔKelch), FLAG-LZTR1^ΔBTB1^ (ΔBTB1), FLAG-LZTR1^ΔBTB2^ (ΔBTB2). **l**, **m** FCM of HLA-ABC expression in *LZTR1* KO HaCaT cells additionally transfected with EV (−), FLAG-LZTR1 (FULL), FLAG-LZTR1^ΔKelch^ (ΔKelch), FLAG-LZTR1^ΔBTB1^ (ΔBTB1), FLAG-LZTR1^ΔBTB2^ (ΔBTB2) and Quantitation (**m**) (*n* = 3). **n** qPCR of HLA-A, HLA-B mRNA expression among *LZTR1* KO HaCaT cells transfected with EV (−), FLAG-LZTR1 (FULL), FLAG-LZTR1^ΔKelch^ (ΔKelch), FLAG-LZTR1^ΔBTB1^ (ΔBTB1), FLAG-LZTR1^ΔBTB2^ (ΔBTB2) (*n* = 3). **o** Mechanistic model of LZTR1 on co-translational biogenesis process. Up: ubiquitination-dependent p50 generation from p105; down: ubiquitination-independent but LZTR1-involved p50 generation from p105. ns: not significant; **P < 0.01, ****P < 0.0001 by one-way ANOVA (**m**, **n**). Data are shown as mean ± SEM.

Our findings indicate that in *Lztr1*-deficient KCs, MHC-I antigen presentation is impaired, blunting reactivation of epidermal CD8^+^ T_RM17_ cells. We next aimed to explore the relationship between LZTR1 and the generation of NF-κB1 P50. Consistent with reports that the proteasome influences degradation of NF-κB1 precursor p105 into active form p50^47,50^, production of NF-κB1 p50 was influenced by MG132, a proteasome inhibitor, but not with the lysosome inhibitor bafilomycin A1 (Baf-A1) (Fig. 8a, b). To gain insights into how LZTR1 regulates the degradation and activation of NF-κB1, we focused on ubiquitin- and proteasome-co-mediated processing of NF-κB1. Cycloheximide (CHX), an inhibitor of eukaryotic translation elongation^51^, and MG132 chasing experiments showed that *LZTR1*-KO slowed the degradation of p105 and generation of p50 in a proteasome-dependent manner (Fig. 8c–f). However, both colocalization of p105 and ubiquitin and immunoprecipitation of p105 experiments revealed that p105 is equally ubiquitinated in the absence of LZTR1 (Supplementary Fig. S14a, b). Notably, we did not observe a direct binding between LZTR1, an adaptor for CUL3 ubiquitin ligase complex^24^, and NF-κB1 p105 (Supplementary Fig. S14c, d), indicating that LZTR1 does not regulate NF-κB1 activity through ubiquitin-mediated p105 degradation.

To determine the interactome of LZTR1 in cells, we performed a mini-TurboID proximity biotinylation-based experiment on YUSIK, a melanoma cell line, based on our preliminary work^25^ (Supplementary Fig. S14e, f), and FLAG tag-based or LZTR1 antibody-based coimmunoprecipitation (co-IP) experiments on HaCaT, followed by liquid chromatography coupled to mass spectrometry (LC-MS). Intriguingly, proteasome and ribosome complexes were highly enriched in precipitates from two cell lines (Supplementary Fig. S14g, h). We identified PSMA6, a reported genetic risk factor for the development of psoriasis and IBD^52,53^, PSMA7 (proteasome 20S subunit alpha), a biomarker of IBD^54^, and RPS3 (ribosome small 40S subunit) as potential LZTR1-interacting proteins (Supplementary Fig. S14i), while only PSMA7 and RPS3 were validated through co-IP (Fig. 8g). We further demonstrate that the critical binding domains of LZTR1 with RPS3 were the BTB1 and BTB2 regions, as the Kelch domain mutant had no influence on LZTR1 binding with RPS3 (Fig. 8h, i).

One study has demonstrated a ubiquitination-independent, but proteasome and ribosome-dependent, manner of NF-κB1 p50 generation through co-translational biogenesis^55^. We hypothesized that LZTR1 participates in the generation of NF-κB1 p50 by acting as a bridge for ribosome and proteasome to undergo proximal and complete co-translational biogenesis process (Fig. 8o), which was confirmed by restrictive colocalization of proteasome and ribosome in the LZTR1^full^ and LZTR1^ΔKelch^ groups (Fig. 8j). In addition, the recovery effect on p50 generation and MHC-I molecular expression was achieved in the LZTR1^full^ and LZTR1^ΔKelch^ groups but not in the LZTR1^ΔBTB1^ and LZTR1^ΔBTB2^ groups (Fig. 8k–n), indicating that the reduction of proteasome interaction with ribosome blunts the co-translational biogenesis of NF-κB1 p50 and modulates the transcription of MHC-I molecules.

## Discussion

In recent years, enormous attention has been paid to the role of T_RM_ cells in various autoimmune diseases such as psoriasis and IBD^7,8,10^. While CD4^+^ T_RM_ cells, historically seen as the primary source of inflammatory cytokines, have garnered considerable focus^11^, the mechanisms by which CD8^+^ T_RMs_ are maintained and activated within tissue, such as the epithelia, have remained elusive. Our study provides critical information on this and identifies LZTR1 as a major component in activating CD8^+^ T_RM_ within both the skin and gastrointestinal epithelium to amplify inflammation in an MHC-I-dependent manner, providing a sharp departure from the conventional pathogenic loop between DC and CD4^+^ T in autoimmune diseases such as psoriasis^13^.

The roles of MHC-I, including HLA-A/B/C, are well-established in tumor biology^56–58^, where IFNγ, secreted by tumor-infiltrating T cells, can induce expression of MHC-I on tumor cells and boost CD8^+^ T cells cytotoxicity^59^. However, the role of MHC class I is less established in cutaneous inflammatory and autoimmune diseases. Here, we identify a novel mechanism in autoimmunity and activation of CD8^+^ T cells, particularly CD8^+^ T_RM_ cells, through modulation of MHC-I expression by LZTR1 in an NF-κB1-dependent manner. The implications of these findings are directly applicable to diseases such as psoriasis, where epidermal CD8^+^ T cells recognize autoantigens presented by MHC-I expressed on the surface of KCs. Furthermore, we extend these insights to encompass IBD (Supplementary Fig. S13), suggesting broader implications for autoimmune therapy. The nature of those antigens has remained unclear, although some studies have proposed endogenous antigens such as the antimicrobial peptide LL37 and keratins such as K16 and K17^18,60–62^, shifting the CD8^+^ T-cell responses from a cytotoxic to pro-inflammatory cytokine response^63,64^. Notably, in line with the mechanisms outlined here, we observed decreased expression levels of these putative autoantigens, including *Camp* (encoding LL37)*, Krt6a, Krt6b*, and *Krt16* in *Lztr1*-deficient KCs (Supplementary Fig. S6k). Taken together, through both the short-term psoriasis model and the rechallenge model, our data identify LZTR1 as a novel driver of pro-inflammatory KC subsets that amplifies autoantigen presentation and promotes the activation of both CD8⁺ T_RM_ and Tc17 cells. In this context, Tc17 cells primarily contribute to the acute inflammatory response, whereas CD8⁺ TRM cells play a central role in psoriasis recurrence. This initiates a self-sustaining, cytokine-driven feedback loop between CD8⁺ T cells and KCs, further enhancing MHC-I expression, as supported by our scRNA-seq data (Fig. 1k).

Another notable finding is the role of LZTR1 in activating and amplifying immune responses on rechallenge, a feature of many, if not most, autoimmune diseases such as psoriasis, where the skin lesions tend to recur at previously affected sites^65^. CD8^+^ T_RM_ cells, which are responsible for long-term skin immunity and react rapidly to antigens, are enriched in the epidermis of inflamed psoriatic skin and contribute to proinflammatory local immune microenvironment^12,13,66,67^, and are found in increased levels in clinically resolved skin^12^, where they can be triggered to reactivate the disease process^68^. Based on our findings from the scRNA-seq and FCM, we reveal impaired inflammatory function of CD8^+^ T_RM_ in *Lztr1*-deficient mouse epidermis. Typically, T memory cells are usually maintained and survive through a combination of IL-7 and IL-15, where they do not need signaling through MHC^69^. However, for reactivation, they require secondary stimulation by peptide-MHC-I complexes^70,71^. Interestingly, this peptide-MHC-I expression depends on LZTR1 function in KCs and is mandatory for CD8^+^ T_RM_ oligoclonal expansion and IL-17A production during immune rechallenge. Our data highlight a promising therapeutic potential target to reverse psoriasis recurrence issues.

Processing of NF-κB1 p105 precursor into its active form p50 requires proteasome and ubiquitin conjugation. However, p50 can also be co-translationally generated in a ubiquitination-independent processing event but involves the 20S/26S proteasome and ribosome, in which p50 is generated from the nascent polypeptide of p105 in an extended and unfolded state^47,55,72^. Here, we show that PSMA7 from proteasome 20S alpha subunit and ribosome small 40S subunit RPS3 interact with LZTR1. In addition, we identify the BTB1 and BTB2 domains of LZTR1 as the functional fragments that promote the interplay between ribosome and proteasome. Hence, these observations suggest that LZTR1 may act as a mediator protein for bringing ribosome and proteasome in proximity to enhance the co-translational biogenesis of p50 (Fig. 8o). Additional work will be required to elaborate on the detailed mechanisms by which LZTR1 works with ribosome and proteasome.

However, our study has some limitations. Due to limitations in establishing in vitro co-culture systems for CD8^+^ T cells and target cells such as KCs in autoimmune settings, we were unable to directly validate the cellular interactions between IL17A^+^CD8^+^ T cells and KCs. Second, future validation and focus on CD8^+^ T_RM_ TCR sequences and identification of the specific self-antigens involved may give rise to a deeper understanding of MHC-peptide/TCR interaction and eventual targeting of the specific antigens.

In summary, our study identifies a novel regulatory role of LZTR1 on epithelial immune homeostasis and activation and maintenance of CD8^+^ T_RM_ function in both skin and gut epithelia. In addition, it provides novel insights into the mechanisms that drive and maintain CD8^+^ T_RM_ responses in epithelial autoimmune diseases. Furthermore, our data highlight MHC-I and LZTR1 as novel therapeutic targets in autoimmune diseases, especially for patients prone to recurrent episodes.

## Materials and methods

### Ethics approval

All human studies were approved by the ethics committee of the Central South University (ethics number: 202308636) and performed in accordance with the Declaration of Helsinki. Written informed consent for sample collection was obtained from all subjects. All animal care protocols and experiments were approved by the Care and Use Committee of the Department of Laboratory Animals.

### Human samples

For IF analysis, we borrowed the paraffin embedded-tissues of patients with psoriasis who were diagnosed by Department of Dermatology after histological analysis and healthy controls who had surgical operations from the Department of Dermatology. For scRNA-seq analysis, 3 *×* 4-mm^2^ skin punch biopsies were obtained from lesional and non-lesional skin from 3 patients with psoriasis. Similarly, a same-size skin was also collected from one healthy donor who had skin flap transplantation as a control skin sample. Detailed clinical information including age, gender, position, and cell number were shown in Supplementary Fig. S1b. Additionally, psoriasis disease activity was assessed using the clinical PASI score as previous described^73^.

### Mice

All wild type (WT) BALB/c and C57BL/6 mice were purchased by Hunan SJA Laboratory Animal Co, Ltd. (Changsha, China). *Lztr1*^fl/fl^ mice were constructed by Cyagen Biosciences Inc. (Guangzhou, China), of which genetic background is C57BL/6 J. Mice with specific deletion of *Lztr1* were generated by crossing *Lztr1*^fl/fl^ mice with K14-Cre or Lyz2-Cre transgenic mice. All mice were housed under a regular 12-h light-dark cycle with free access to water and food and under specific pathogen-free conditions. The temperature was 22 ± 1 °C. Licenses for breeding and experiment were obtained from the Department of Laboratory Animals of University. All mice used in this study were 8–12 weeks old, controls were age- and gender-matched littermates.

### IMQ-induced psoriasis mouse model

To induce acute psoriasis-like skin inflammation, IMQ cream was topically applied to ear or back skin of female mice. In brief, the mice were treated topically on their ventral sides of ear (15 mg per ear) or shaved back (62.5 mg per mouse) with IMQ cream for 6 consecutive days (from day 0 to day 5) and sacrificed on day 6. For the recurrent disease model of psoriasis, the *Lztr1*^fl/fl^ and K14^cre^*Lztr1*^fl/fl^ mice were subjected to a daily topical dose of 62.5 mg IMQ cream for 6 consecutive days, after 21 days of recovery, both groups of mice were rechallenged with secondary application of same doses of IMQ for 6 days and sacrificed on day 31. PASI score was recorded daily and used to monitor and evaluate the skin phenotype. Erythema, thickness, and scaling of lesional skin were scored independently from 0 to 4 (0, none; 1, slight; 2, moderate; 3, marked; 4, severe). The cumulative score of three parameters was used as a measure of the severity of skin inflammation (scale 0–12). Ear thickness was measured daily with a digital vernier caliper (AIRAJ). Spleen index was defined as the ratio of spleen weight (mg) to body weight (g) of mouse.

### DSS-induced IBD mouse model

Male mice aged 8–10 weeks were randomly dived into two groups based on body weight and genotype. To induce acute colitis mouse model, DSS (cat. 160110, MP Biomedicals) was added to the mice drinking water at 2% (w/v) ad libitum for 6 days. Control group received sterilized water ad libitum. In the modeling period, the weight loss, fecal occult blood and stool consistency of all mice were daily assessed and recorded to score the DAI as previously described^74^. For assessment of intestinal pathology, colons were fixed and stained with hematoxylin and eosin (H&E), the degree of inflammation (0, none; 1, slight; 2, moderate; 3, marked; 4, severe), the distribution of damage (0, none; 1, single; 2, multiple; 3, nearly diffuse; 4, diffuse), and the infiltration of inflammation (0, none; 1, mucosa; 2, mucosa and submucosa; 3, limited transmural; 4, transmural) were scored independently and accumulated to evaluate the severity of pathology.

### Administration of CD4 and CD8 depletion antibodies

CD4 (cat. A2101) and CD8α (cat. A2102) antibodies for cell depletion and IgG2a (cat. A2117) for isotype control were all purchased from Selleck. Anti-CD4 and anti-CD8α were administered either in combination or as single agent by intraperitoneal injection in doses of 100 ug per antibody per mouse on day 1 and day 4 before IMQ or DSS induction, same volume of IgG was injected into control group.

### Intracutaneous injection of AAV in mouse ear

AAV2 vector-mediated excessive H2-D1 expression and scrambled control (EGFP) were constructed and purified by Beijing Syngentech Co., Ltd. (Beijing, China). A total 50 μL of 1 *×* 10^12^ vg/mL of AAV2 H2-D1 diluted in phosphate-buffered saline (PBS) was intracutaneously injected into the ventral side of the right ear, the contralateral ear of mouse was received the same volume of PBS containing 1 *×* 10^12^ vg/mL AAV2 as a control. After 7 days of the injection, mice were treated with IMQ for 6 days and then sacrificed for further analysis.

### Histopathology, immunohistochemistry, and immunofluorescence

For histopathology analysis, mouse ear, lung, colon, and dorsal skin were harvested and fixed in 4% paraformaldehyde (PFA) at least 24 h followed by dehydrated and embedded in paraffin. 5–7-μm sections were stained with H&E according to standard procedures. For immunostaining analyses of mouse and human tissues, the sections were deparaffinized and stained with antibodies against PCNA (cat. ab15497, Abcam, 1:200), LZTR1 (cat. ab106655, Abcam, 1:100), K10 (cat. ab76318, Abcam, 1:2500), K14 (cat. ab181595, Abcam, 1:2000), K14 (cat. ab7800, Abcam, 1:1000), CD8α (cat. 66868-1-Ig, Proteintech, 1:400), CD8α (cat. ab209775, Abcam, 1:2000), HLA-C (cat. PA5-79367, Thermo Fisher, 1:500), IL-17A (cat. ab79056, Abcam, 1:100).

In detail, tissue samples were stained for 3 h at 60 °C, and then deparaffinized. Antigen was retrieved at EDTA antigen retrieval buffer (pH 8.0) and maintained at a sub-boiling temperature for 15 min, allowed to cool down at room temperature (RT) for 8 min, and then reheated at sub-boiling temperature for an additional 8 min. The samples were then blocked in 3% BSA, PBS with 0.25% Triton X-100 for 1 h at room temperature.

For IF, after incubated with first antibody overnight at 4 °C in the blocking solution, the secondary antibody including Alexa Fluor 594-conjugated goat anti-mouse IgG (cat. A21203, Thermo Fisher, 1:1000), Alexa Fluor 488-conjugated donkey anti-rabbit IgG (cat. A21206, Thermo Fisher, 1:1000), Alexa Fluor 594-conjugated goat anti-rabbit IgG (cat. A21207, Thermo Fisher, 1:1000), or Alexa Fluor 488-conjugated donkey anti-mouse IgG (cat. A21202, Thermo Fisher, 1:1000) was added to the blocking solution and incubated for 2 h. Then, the slides were covered with anti-fade mounting medium with DAPI (cat. ab104139, Abcam) and slips at RT for 10 min, kept in dark place. Images were detected and captured by Fluorescent Microscopy (Nikon, ECLIPSE Ts2R).

For immunohistochemical, after incubated with primary antibodies as indicated, each tissue slide was stained with biotin-conjugated secondary antibody and then incubated with an avidin-biotin-peroxidase complex. Visualization of the target protein was performed by diaminobenzidine (DAB), where the presence of brown color indicates the expression of the targeted molecule. To quantify these immunohistochemical results, the images were further processed by ImageJ.

### Confocal assay

HaCaT cells were fixed with 4% PFA for 15 min, permeabilized with 0.1% Triton X-100 for 30 min, and blocked with 3% BSA for 30 min at RT. Cells were stained with the indicated primary antibodies: anti-RPS3 (cat. ab128995, Abcam, 1:200), anti-Proteasome 20S α (cat. ab22674, Abcam, 1:500), anti-NF-κB1 p105/p50 (cat. 13586S, CST, 1:200), anti-Ubiquitin (cat. sc-8017, Santa Cruz, 1:50), overnight at 4 °C, and then incubated with Alexa Fluor 594-conjugated goat anti-mouse IgG (cat. A21203, Thermo Fisher, 1:1000), Alexa Fluor 488-conjugated donkey anti-rabbit IgG (cat. A21206, Thermo Fisher, 1:1000), Alexa Fluor 594-conjugated goat anti-rabbit IgG (cat. A21207, Thermo Fisher, 1:1000), and Alexa Fluor 488-conjugated donkey anti-mouse IgG (cat. A21202, Thermo Fisher, 1:1000) for 50 min at room temperature. DAPI (cat. ab104139, Abcam) was used to label cell nuclei. Fluorescence was detected using ZEISS LSM 900.

### Cell culture and treatment

The human immortal KC cell line HaCaT was cultured in RPMI 1640 medium supplemented with 10% fetal bovine serum (FBS); 293 T cell line was grown in DMEM/high glucose containing 10% FBS. To isolate primary KCs, the discarded foreskin biopsy tissues, or the skin of newborn pups (1–3 days of age) were collected. After subcutaneous adipose tissues were removed, skin tissues were digested in RPMI 1640 medium containing Dispase II (2 mg/mL) for overnight at 4 °C. The next day, epidermis was isolated and then digested into a single-cell suspension with 0.05% trypsin-EDTA for 10 min at 37 °C. Cells were seeded at the appropriate density. Primary KCs were cultured in the serum-free medium supplement with indicated growth factors (cat. 192060, Lonza, CH) and subcultured according to the cell fusion. All cells were cultured at 37 °C and 5% CO_2_.

For cell stimulation experiments, KCs were treated with the recombinant human IL-17A (200 ng/mL, cat. 7955-IL-100/CF, R&D), TNFα (10 ng/mL, cat. 210-TA-005, R&D), 5 μM QNZ (cat. S4902, Selleck), 1 ng/mL IFN-γ (cat. 300-02, Peprotech) for 24 h according to respective experiment. Additionally, 20 μM MG132 (cat. S2619, Selleck), 400 nM Baf-A1 (cat. S1413, Selleck), and 50 μg/mL CHX (cat. S7418, Selleck) were used to stimulate HaCaT cell line for indicated times.

### Isolation and activation of primary T cells

Spleens were isolated from 8-week sterilized female C57BL/6 J mice on a clean bench, and were pushed through 40-μM cell strainers to obtain suspended single cells. After lysed, erythrocytes were counted and cell viability was calculated. According to the manufacturer’s instructions, CD4^+^ and CD8^+^ primary T cells were separately identified using Dynabead FlowComp Mouse CD4 (cat.11461D, Thermo Fisher Scientific) and Dynabead FlowComp Mouse CD8 (cat.11462D, Thermo Fisher Scientific). Both CD4^+^ and CD8^+^ primary T cells were cultured in RPMI 1640 supplemented with 10% FBS (cat. 10099141c, Gibco) and stimulated with Dynabeads™ Mouse T-Activator CD3/CD28 Kit (cat. 11452D, Thermo Fisher) combined with 20 ng/mL IL-2 (cat. 51061-MNAE, Sino Biological) for 3 days. The activation of T cell was detected by FCM with CD44 and CD62L expression.

### T cell differentiation assay

The Dynabeads in T cells suspension were removed by DynaMag™-2 (cat. 12321D, Thermo Fisher). The activated T cells were washed by Dulbecco’s Phosphate-Buffered Saline (DPBS), and then cultured in RPMI 1640 supplemented with 10% FBS with 20 ng/mL IL-2 (cat. 51061-MNAE, Sino Biological), 20 ng/mL IL12 (cat. 210-12, Peprotech), 5 μg/mL anti-CD3 (cat. 16-0031-85, Thermo Fisher), 2 μg/mL anti-CD28 (cat. 16-0281-82, Thermo Fisher) and 10 μg/mL anti-IL4 (cat. 16-7041-85, Thermo Fisher) for another 3 days. CD4^+^ and CD8^+^ primary T cells were collected and IFNγ expression was detected by FCM.

### siRNA and plasmids information

For RNA interference, cells with 70% confluence were transfected with siRNA (purchased from Genepharma, Shanghai, China) against RELA, NFKB1, NFKB2, or vehicle using Lipofectamine 2000 Transfection Reagent (cat. 11668019, Thermo Fisher, USA) according to the protocol. The targeting sequences were described as follows: human siRELA: AACAGTGTGTCATCCTTCT, human siNFKB1: TAGTCTACATTTGAGACCG, human siNFKB2: ACAAATACGTGTAGACACC, mouse siNFKB1: TATAACTCACTCAGTTTCG. To knockout *LZTR1* in human KCs, sg*LZTR1* plasmid was constructed with lentiCRISPR v2 vector. For CRISPR sg*LZTR1* sequences: forward, CACCGCCATGGGCTGACCTAGCGAC, reverse, AAACGTCGCTAGGTCAGCCCATGGC. All these human oe-*NFKB1*, HA-RPS3, oe-*LZTR1*, and fragment-depleted plasmids were synthesized by Beijing Syngentech. Specific fragment depletion of LZTR1 was designed and shown in Fig. 8h.

### CCK-8 assay

The ability of cell proliferation was examined by cell counting kit-8 (CCK-8) assay (cat. B34302, Selleck). Briefly, HaCaT or primary KCs were planted in 96-well plates (2500 cells/well) and examined every 24 h. 10 µL of CCK-8 solution and 100 µL medium were added into each well and measured spectrophotometrically at 450 nm after 2 h of incubation.

### Flow cytometry

Mouse lymph nodes and spleen were mechanically dissociated to obtain single-cell suspensions, and splenic cells and PBMC were treated with RBC lysis buffer to deplete red blood cells. Intestinal epithelial cells (IECs) were extracted from *Lztr1*^fl/fl^ and K14^cre^*Lztr1*^fl/fl^ mice; briefly, the colons were cut and divided into 1-cm pieces, and incubated in RPMI 1640 containing 10 mM HEPES, 5 mM EDTA and 1.0 mM DTT for 20 min at 37 °C. The remaining colons were then suspended in RPMI 1640 supplemented with 20 mM HEPES and 5 mM EDTA at 37 °C for 30 min. After incubation, the remnant intestinal tissue was removed, IECs were centrifuged and collected at 2000 rpm for 20 min at 4 °C. For ear or back skin of single-cell suspensions, tissues were collected and digested with 2 mg/mL Dispase Ⅱ (cat. D4693, Sigma) in RPMI 1640 medium for overnight at 4 °C to isolate epidermis and dermis. Next, epidermis was digested into a single-cell suspension with 0.05% trypsin-EDTA while dermis was mechanically disrupted and treated with 2 mg/mL Collagenase Ⅳ (cat. V900893, Sigma) in RPMI 1640 for 1 h at 37 °C before being filtered with 70-μm strainers. For cell surface staining, cells were stained with antibodies against membrane antigens on ice for 40 min. Viability dye (cat. 564997, Horizon™ Fixable Viability Stain 700, BD; cat. 423102, Zombie Aqua Fixable Viability Kit, Biolegend) was used to exclude dead cells. For intracellular cytokine staining, cells were stimulated with Cell Stimulation Cocktail (plus protein transport inhibitors) (cat. 00-4975-93, eBioscience) at 37 °C for 6 h. After surface labeling, cells were fixed and permeabilized with FOXP3/Transcription Factor Staining Buffer overnight (cat.00-5523-00, eBioscience) followed by intracellular staining with antibodies against cytokines. The following antibodies were from Biolegend: anti-mouse CD45 (cat. 103116), anti-mouse CD3 (cat. 100328), anti-mouse CD3 (cat. 100228), anti-mouse TCRβ (cat. 109243), anti-mouse CD4 (cat. 100406), anti-mouse CD8a (cat. 100722), anti-mouse IFN-γ (cat. 505810), anti-mouse IL-17A (cat. 506904), anti-mouse IL-17A (cat. 506927), anti-mouse TNF-α (cat. 506306), anti-mouse PD-1 (cat. 135218), anti-mouse CTLA-4 (cat. 106323), anti-mouse FOXP3 (cat. 126404), anti-mouse CD44 (cat. 103012), anti-mouse CD62L (cat. 104407), anti-mouse CD103 (cat. 121422), anti-mouse GZMB (cat. 372207), anti-mouse CD11c (cat. 117306), anti-mouse CD11b (cat. 101216), anti-mouse I-A/I-E (cat. 107625), anti-mouse Ki-67 (cat. 151212), anti-mouse Ly-6G (cat. 127628), anti-mouse F4/80 (cat. 123116), anti-mouse H-2K^d^/ H-2D^d^ (cat. 114714), anti-human HLA-DR (cat. 327021). The following antibodies were from BD pharmingen: anti-mouse TCRγ (cat. 553177), anti-mouse CD4 (cat. 562891), anti-mouse CD8a (cat. 563068). Anti-mouse IL-22 (cat. 17-7222-82), anti-mouse H-2Kb (cat. 11-5958-82), and HLA-ABC monoclonal antibody (cat. MA511723) were purchased from Thermo Fisher. Goat anti-mouse IgG/PE (cat. abs20007, absin) was used to label the membrane HLA-ABC indirectly. Data were obtained from BD FACS LSRFortessa flow cytometer and analyzed using the FlowJo software. The gating strategy was listed in Supplementary Fig. S15.

### RNA isolation and qPCR

Fresh skin samples were collected and flash-frozen in liquid nitrogen, and frozen tissues were fully broken with a homogenizer. Total RNA was extracted from cells or pretreated tissues using MagZol reagent (cat. R4801-01, Magen, Guangzhou, China). The synthesis of cDNA (cat. 11141ES60, Yeasen, Shanghai, China) and qPCR (cat. B21703, Bimake, USA) were performed according to the manufacturer’s instructions. According to the manufacturer’s instructions, each component was added to the reaction system (20 μL) and qRT-PCR was performed using a on QuantStudio-3 Real-Time PCR system (Thermo Fisher Scientific, Waltham, MA, USA). Related gene-specific primers are listed in Supplementary Table S1.

### Immunoprecipitation and immunoblotting

For NF-κB1 immunoprecipitation, we collected the HaCaT cells with LZTR1 depletion or control and extracted protein using NP-40 lysis buffer (cat. P0013F, Beyotime) supplemented with protease and phosphatase inhibitors (PPI) (cat. B14002, B15002, Bimake). The lysates were incubated with anti-NF-κB1 (cat. 13586S, CST, 1:100) or rabbit IgG (cat. A7016, Beyotime, China, 1:100) for overnight at 4 °C, followed by binding of protein A/G magnetic beads (cat. 88802, Thermo Fisher) based on the manufacturer’s instructions. The immunoprecipitates were then subjected to immunoblotting (IB). In addition, HaCaT cells treated with or without FLAG-LZTR1 transfection were used for FLAG immunoprecipitation using the Flag-tag Protein IP Assay Kit with Magnetic Beads (cat. P2181S, Beyotime) according to the protocols. For IB, cells or mouse skin tissues were lysed by RIPA containing PPI. Protein concentrations were measured with the BCA reagent (Beyotime, China) by using a Beckman Coulter DU-800 spectrophotometer. Equal amounts of protein were resolved by SDS–PAGE and immunoblotted with anti-LZTR1 (cat. ab289965, Abcam, 1:1000), anti-HLA-A (cat. ab52922, Abcam, 1:1000), anti-HLA-B (cat. ab193415, Abcam, 1:1000), anti-HLA-C (cat. sc-166057, Santa Cruz, 1:100), anti-GAPDH (cat. 60004-I-Ig, Proteintech, 1:3000), anti-HLA-C (cat. PA5-79367, Thermo Fisher, 1:1000), anti-NFκB1 p105/p50 (cat. 13586S, CST, 1:1000), anti-NFκB2 p100/p52 (cat. 37359S, CST, 1:1000), anti-NFκB p65 (cat. 8242S, CST, 1:1000), anti-pho-NFκB p65 (cat. 3033S, CST, 1:1000), anti-c-REL (cat. ab133251, Abcam, 1:1000), Anti-Erk (cat. 4695S, CST, 1:1000), anti-p-Erk (cat. 4370S, CST, 1:1000), anti-P38 (cat. 8690S, CST, 1:1000), anti-p-P38 (cat. 4511S, CST, 1:1000), anti-TUBULIN (cat. 11224-1-AP, Proteintech, 1:2000), anti-FLAG (cat. F1804-1MG, Sigma, 1:1000), anti-HA-Tag (cat. sc-7392, Santa Cruz, 1:100), anti-PSMA6 (cat. ab109377, Abcam, 1:50000), anti-PAMS7 (cat. ab133502, Abcam, 1:1000), anti-RPS3 (cat. ab128995, Abcam, 1:1000), anti-Ubiquitin (cat. sc-8017, Santa Cruz, 1:100). HRP goat anti-mouse IgG (H + L) (cat.AS003, Abclonal, 1:5000), and HRP goat anti-rabbit IgG (H + L) (cat. AS014, Abclonal, 1:5000) were used as secondary antibodies. The immunoblots were detected using a gel image analysis system (LI-COR, USA).

### Luciferase reporter assay

For promoter reporter assay, we designed 2 separate fragments for upstream 1000 bp sequence from the TSS of *HLA-A* or *HLA-B* respectively, including P1 (from –1000 bp to –501 bp, pGL3-P1) and P2 (from –500 bp to –1 bp, pGL3-P2). We also conducted fragment P2 with a mutant NF-κB1-binding sequence for *HLA-A* or *HLA-B,* respectively (prom. MUT), which were all subcloned into the pGL3-basic-SV40-hRluc dual promoter vector (Beijing Syngentech). The designed diagrams were presented in Fig. 7p, q, t. HaCaT cell line in 24-well plates was transfected with HLA-A or HLA-B luciferase reporter plasmid (1 μg) using Lipofectamine 2000 Transfection Reagent according to the protocol. Firefly and Renilla luciferase activity was measured using the Dual Luciferase Reporter Assay System (cat. E1910, Promega, USA). The ratio of firefly luciferase activity to Renilla luciferase activity was calculated.

### ChIP-qPCR

ChIP was performed using SimpleChIP® Enzymatic Chromatin IP Kit (cat. 9003S, CST) according to the manufacturer’s instruction. 10 μg anti-NF-κB1 (cat. 13586S, CST, 1:200) or the negative control IgG was incubated for overnight at 4 °C. SYBR Green qPCR was performed using the human HLA-A primers: forward, AAGGCGGTGTATGGATTG; reverse, CTGATTGGCTTCTCTGGAA; HLA-B primers: forward, CTTCCAGGATACTCGTGAC; reverse, CGCTGATTGGCTTCTCTA. The signals were expressed as a percentage of the total input chromatin.

### Proximity labeling

YUSIK cell, a primary human melanoma cell, was transiently transfected with pIND-LZTR1-HA-mini-TurboID plasmid, which was induced by doxycycline (100 ng/mL, 24 h) stimulation or not and treated with 50 μM biotin for 1 h prior to harvesting in RIPA lysis buffer. Lysates were then incubated with streptavidin agarose beads overnight at 4 °C with rotation. Proteins were eluted by heat denaturation followed by MS analyses.

### Protein MS

Samples from mouse skin tissue and immunoprecipitates pulled down with anti-LZTR1 or anti-FLAG (LZTR1 exogenous) were analyzed through MS. The proteomic profiling was conducted by PTM Bio (China). Following the instructions, the sample was pulverized with liquid nitrogen and transferred to a 5-mL centrifuge tube. Four volumes of lysis buffer (8 M urea, 1% protease inhibitor cocktail, and phosphatase inhibitor) were added to the tissue powder. The mixture underwent three cycles of sonication on ice using a high-intensity ultrasonic processor (Scientz). Subsequent centrifugation at 12,000*×* *g* and 4 °C for 10 min removed remaining debris. The supernatant was collected, and protein concentration was determined with a BCA kit following the manufacturer’s instructions.

For protein digestion, the protein solution was first reduced with 5 mM dithiothreitol at 56 °C for 30 min and then alkylated with 11 mM iodoacetamide at RT in darkness for 15 min. The protein sample was diluted by adding 100 mM TEAB to achieve a urea concentration of less than 2 M. Trypsin was applied at a 1:50 trypsin-to-protein mass ratio for an initial overnight digestion, followed by a second 4-h digestion at a 1:100 trypsin-to-protein mass ratio. The resulting peptides were desalted using a C18 solid-phase extraction (SPE) column.

For TMT labeling, tryptic peptides were initially dissolved in 0.5 M TEAB. Each set of peptides was individually labeled with the respective TMT reagent according to the manufacturer’s protocol (ThermoFisher Scientific) and incubated for 2 h at RT. A 5% hydroxylamine solution was used to quench the labeling reaction. The pooled samples were desalted using a Strata X C18 SPE column (Phenomenex) and dried via vacuum centrifugation.

MS analysis was performed utilizing a Q ExactiveTM HF-X mass spectrometer (ThermoFisher Scientific) with a nano-electrospray ion source. The electrospray voltage was set at 2.0 kV. Full MS scans had a resolution of 60,000 within the 350–1600 m/z range. Up to 20 of the most abundant precursor ions were selected for subsequent MS/MS analysis, with a 30-s dynamic exclusion. High-energy collision dissociation (HCD) fragmentation was conducted at a normalized collision energy (NCE) of 28%, and resulting fragments were detected in the Orbitrap at a resolution of 30,000. The fixed first mass was 100 m/z. AGC target was set at 1E5, with an intensity threshold of 3.3E4 and a maximum injection time of 60 ms. Farther bioinformatics enrichment analyses were conducted through R and R studio.

### scRNA-seq

The fresh skin biopsy tissues of psoriasis patients were stored in the MACS Tissue storage solution (cat.130-100-008, Miltenyi Biotec) on ice after the surgery within 30 min.

For human or mouse skin lesions, the single-cell suspensions were conducted using the same method as described for FCM above. CD45-sorted skin single-cell suspensions were stained with 7-AAD (cat. 51-68981E, BD Phamingen) and CD45 antibody (cat. 103116, Biolegend) and sorted (BD FACSymphony™ S6 Cell Sorter) before proceeding scRNA-seq platform. For human (epidermis) and mouse (epidermis & dermis) total single-cell suspension samples without sorting, we used Dead Cell Removal Kit (cat. 130-090-101, Miltenyi Biotec) to improve cell viability to meet the requirements of scRNA-seq.

Single-cell suspensions (1 × 10^5^ cells/mL) with PBS were loaded into microfluidic devices using the Singleron Matrix® Single Cell Processing System (Singleron Biotechnologies, Koln, Germany). Subsequently, the scRNA-seq libraries were constructed according to the protocol of the GEXSCOPE® Single Cell RNA Library Kits (Singleron)^75^. Individual libraries were diluted to 4 nM and pooled for sequencing. Finally, the pools were sequenced using an Illumina HiSeq X (Illumina, San Diego, CA, USA) with 150-bp paired-end reads.

### scRNA-seq data processing

The FASTQ files were processed using the SCOPE-tools (https://github.com/SingleronBio/SCOPE tools). In this process, the cellular barcode was extracted and corrected, reads were aligned to the reference genome (GRCh38), and gene expression quantification was performed. The resulting unique molecular identifier (UMI) matrix was converted into Seurat objects using the Seurat package (version 4.3)^76^.

### scRNA-seq analysis

For the human or mouse dataset, we removed a cell according to the following criteria: (1) fewer than 200 expressed genes; (2) more than 10% mitochondrial counts; (3) more than 5500 expressed genes. According to our team’s established analysis protocol, single-cell gene expression data were normalized using the “LogNormalize” method with a scale factor of 10,000. Then, the top 2000 most variable genes were identified, and a linear scaling method was applied to standardize the range of expression values for each gene. Principal component analysis (PCA) was performed to reduce dimensionality by “RunPCA” function. After finishing UMAP for dimension reduction on cells, all cell clusters were identified in line with the expression of individual marker genes by using the “FindAllMarker” function.

For further analysis of the characteristic discrepancy of different cell clusters, the “FindAllMarker” function in the Seurat package was used to detect DEGs in the individual clusters according to the Wilcoxon rank test. We define genes with *P*_val < 0.05, avg_log2FC > 1 as upregulated genes and genes with *P*_val < 0.05, avg_log2FC < –1 as down-regulated genes. Gene Ontology, Reactome and KEGG pathway enrichment analyses were performed using the clusterProfiler package (V4.8.3)^77^. The annotation R package org.Hs.eg.db (human) or org.Mm.eg.db (mouse) was used to map gene identifiers. Moreover, we also did GSEA of the enrichment pathway using the clusterProfiler package. A cutoff Q value < 0.05 was applied to select the most significantly enriched pathways.

The “AddModuleScore” function was used to calculate module scores for different pathways. Specifically, the T_RM17_ differentiation score was defined as the average expression of IL17A, IL17F, CCR4, CCR6, ICOS, RORC, TOX, TCF7, MAF, IL22, ITGAE, CXCR6, LAG3 and PDCD1. The cytokine secretion score was defined as the average expression of GZMA, GZMB, GZMH, NKG7, CCL5, GNLY, CST7, TCF7, IFNG, and RPF1. The cytotoxicity score was defined as the average expression of GZMA, GZMB, GZMH, NKG7, CCL5, GNLY, CST7, TCF7, IFNG, and RPF1. The MHC class 1 antigen presentation score was defined as the average expression of Hspa5, Canx, Calr, Tap2, B2m, H2-D1, and H2-K1. The T17 differentiation score was defined as the average expression of Il17a, Il17f, Il22, Ccr6, Il23r, and Rorc. Wilcoxon rank test was used to perform significance tests using the ggpubr package (V0.6.0).

The CellChat (https://github.com/sqjin/CellChat) was used to deduce cell interactions based on known ligand-receptor pairs expressed in various cell types^78^. The official workflow was followed, which involved loading the normalized count matrix into CellChat, preprocessing functions such as “identifyOverExpressedGenes”, “identifyOverExpressedInteractions”, and “projectData” were applied, with standard parameters set. In primary analyses, the core functions “computeCommunProb”, “computeCommunProbPathway”, and “aggregateNet” were examined using standard parameters and a fixed randomization seed.

The cell pseudotime trajectories were reconstructed using the R package monocle 2 (V2.28.0)^79^. Specifically, highly variable genes were identified using the “VariableFeatures” function in the Seurat package, and then cells were sorted onto a proposed temporal trajectory based on highly variable genes.

Furthermore, we applied GSVA method to calculate the hallmark score of individual cells, as implemented in the GSVA package (V1.48.3)^80^ and AUCell package (V1.24.0)^19^.

scRNA-seq data of published healthy and psoriasis human skin were derived from GSE150672, and processed following the same quality control and preprocessing steps as described previously.

### Spatial transcriptomics

Healthy skin and psoriasis patient skin spatial-seq data were from our previously published dataset GSE225475. Space Ranger software (10x Genomics) was used to process sequencing output and the histology images. The reads were aligned to the human genome (hg38). Seurat was then used to analyze the expression matrix, and SCTransform function was used to scale the expression data.

To project the lymphocyte subtypes obtained from our scRNA-seq data to the spatial transcriptomic (ST) data, ST data were deconvolved by lymphocyte subpopulations through RCTD with “doublet” model^81^. Spots in RCTD results labeled as “reject” were recorded as “None”. For the other spots, the proportion of lymphocyte subpopulations was considered as the celltype composition for those spots.

The Euclidean distances between CD8^+^ T_RM17_ spots and LZTR1-high spots (those with LZTR1 expression levels above the upper quartile) were subsequently calculated:

$${\rm{d}}\left({\rm{q}},{\rm{p}}\right)=\sqrt{{\left({q}_{x}-{p}_{x}\right)+\left({q}_{y}-{p}_{y}\right)}^{2}}$$

Here, $({q}_{x},{q}_{y})$ and $({p}_{x},{p}_{y})$ represent the spatial coordinates of CD8^+^ T_RM17_-positive spots and LZTR1-high spots, respectively. Student’s *t*-test was employed to compare the spatial distribution differences between healthy and psoriatic samples.

### Quantifications, statistical analyses, and renderings

FlowJo v10.8.1 software was used for flow cytometric analysis. Image J was used for immunohistochemistry and IF staining analysis. Image Studio was used for IB analysis. Data are presented as mean ± SEM and analyzed using the GraphPad Prism 9 with the paired Student’s *t*-test, unpaired Student’s *t*-test, One-way ANOVA, or Two-way ANOVA. For scRNA-seq data statistical analysis, Wilcoxon rank test was used to compare two groups. A simple linear regression model was used to analyze the correlation between *LZTR1* and *HLA-A*, *HLA-B, HLA-C* mRNA expression. All experiments were repeated three or more times. *P* value less than 0.05 was considered significant, statistical significance was defined as *P < 0.05, **P < 0.01, ***P < 0.001, ****P < 0.0001.

## Supplementary information

Supplementary Information

## Data Availability

All data are available in the main text or the supplementary materials. The scRNA-seq and bulk RNA-seq data reanalyzed in this paper are accessible at the Gene Expression Omnibus under accession numbers GSE150672, GSE13355 and GSE14905, respectively. All other data are available from the corresponding author upon request.
