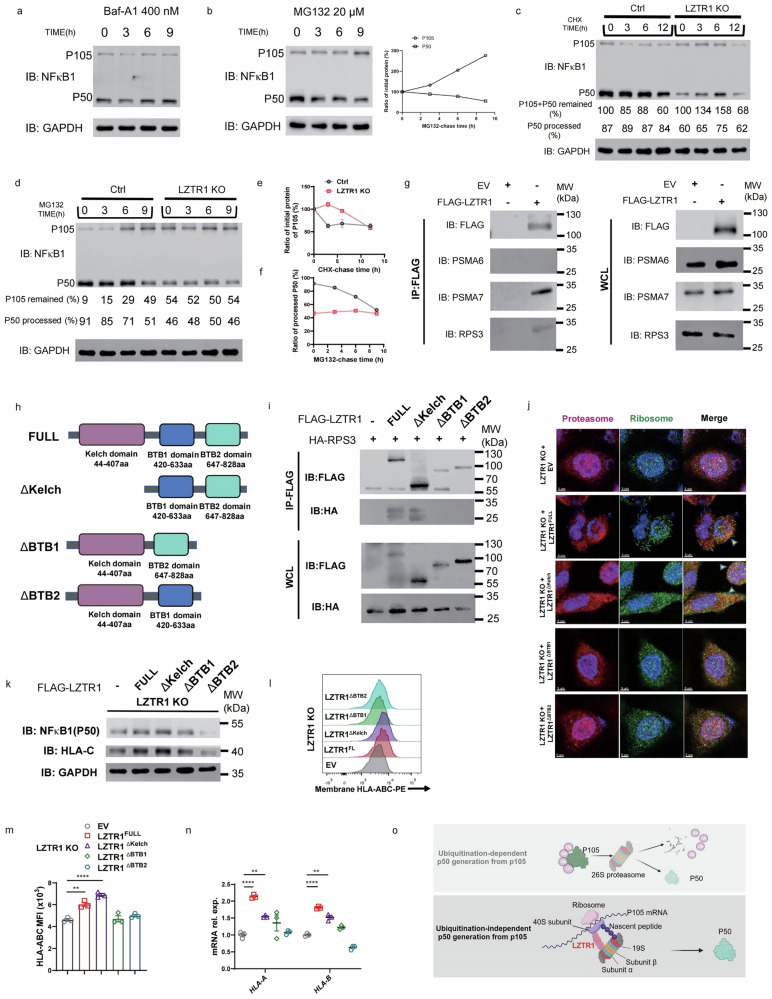# Author Correction: LZTR1 regulates epithelial MHC-I expression via NF-κB1 to modulate CD8^+^ T cells activation

**DOI:** 10.1038/s41421-026-00917-1

**Published:** 2026-07-16

**Authors:** Rundong Jiang, Zhiqin Fang, Yutong Wang, Bo Huang, Junkun Liu, Lam C. Tsoi, Rachael Bogle, Zongbo Zhang, Yehong Kuang, Xin Li, Liang Dong, Liping Jin, Johann E. Gudjonsson, Mingzhu Yin, Xiang Chen

**Affiliations:** 1 Department of Dermatology, Hunan Engineering Research Center of Skin Health and Disease, Hunan Key Laboratory of Skin Cancer and Psoriasis, Xiangya Hospital, Central South University, Changsha, Hunan China; 2 National Engineering Research Center of Personalized Diagnostic and Therapeutic Technology, Central South University, Changsha, Hunan China; 3 Department of Dermatology, University of Michigan, Ann Arbor, MI USA; 4 Clinical Medicine Eight-Year Program, Xiangya School of Medicine, Central South University, Changsha, Hunan China; 5 Clinical Research Center, Medical Pathology Center, Cancer Early Detection and Treatment Center and Translational Medicine Research Center, Chongqing University Three Gorges Hospital, Chongqing University, Wanzhou, Chongqing China

**Keywords:** Autoimmunity, Mechanisms of disease, Transcription

Correction to: *Cell Discovery* 10.1038/s41421-025-00837-6, published online 29 October 2025

In the originally published version of this article, the GAPDH loading control panel in Fig. 8b was inadvertently misplaced during figure assembly. This panel has now been replaced with the correct image derived from the original experimental data. This correction does not affect the statistical results, interpretation of the data, or the conclusions of the study.

Incorrect fig 8:
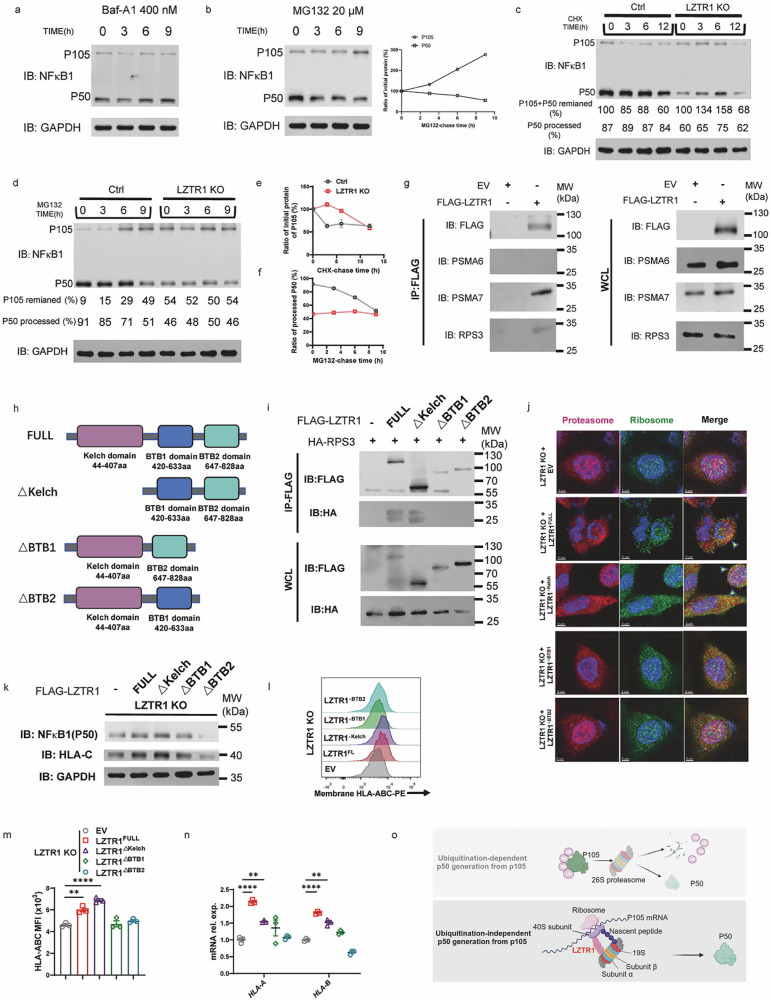


Correct fig 8: