# Supplementary material for: LZTR1 regulates epithelial MHC-I expression via NF-κB1 to modulate CD8+ T cells activation
Source: Cell Discov. 2025 Oct 29;11:84. doi: 10.1038/s41421-025-00837-6 (PMC12572372; doi:10.1038/s41421-025-00837-6)
Supplement: Supplementary file 1 — Supplementary Information [file 41421_2025_837_MOESM1_ESM.pdf]

Supplementary Information

LZTR1 regulates epithelial MHC-I expression via NF-κB1 to modulate $CD8^+$ T cells activation

Rundong Jiang[1,3,4#], Zhiqin Fang[1,3#], Yutong Wang[1,3,5], Bo Huang[1,3], Junkun Liu[1,3], Lam C. Tsoi[4], Rachael Bogle[4], Zongbo Zhang[2], Yehong Kuang[1,3], Xin Li[1,2,3], Liang Dong[1,3], Liping Jin[1,3], Johann E Gudjonsson[4*], Mingzhu Yin[1,2,3*], Xiang Chen[1,3*]

[1]Department of Dermatology, Hunan Engineering Research Center of Skin Health and Disease, Hunan Key Laboratory of Skin Cancer and Psoriasis, Xiangya Hospital, Central South University, Changsha, Hunan, China
[2]Clinical Research Center, Medical Pathology Center, Cancer Early Detection and Treatment Center and Translational Medicine Research Center, Chongqing University Three Gorges Hospital, Chongqing University, Wanzhou, Chongqing, China
[3]National Engineering Research Center of Personalized Diagnostic and Therapeutic Technology, Central South University, Changsha, Hunan, China
[4]Department of Dermatology, University of Michigan, Ann Arbor, MI, USA
[5]Clinical Medicine Eight-Year Program, Xiangya School of Medicine, Central South University, Changsha, Hunan, China

[#]These authors contributed equally to this work.

[*]Correspondence: chenxiangck@126.com (X.C.) or yinmingzhu2008@126.com (M.Y.) or johanng@med.umich.edu (J.E.G.).

The file includes:

Supplementary Figs S1 to S15
Supplementary Table S1

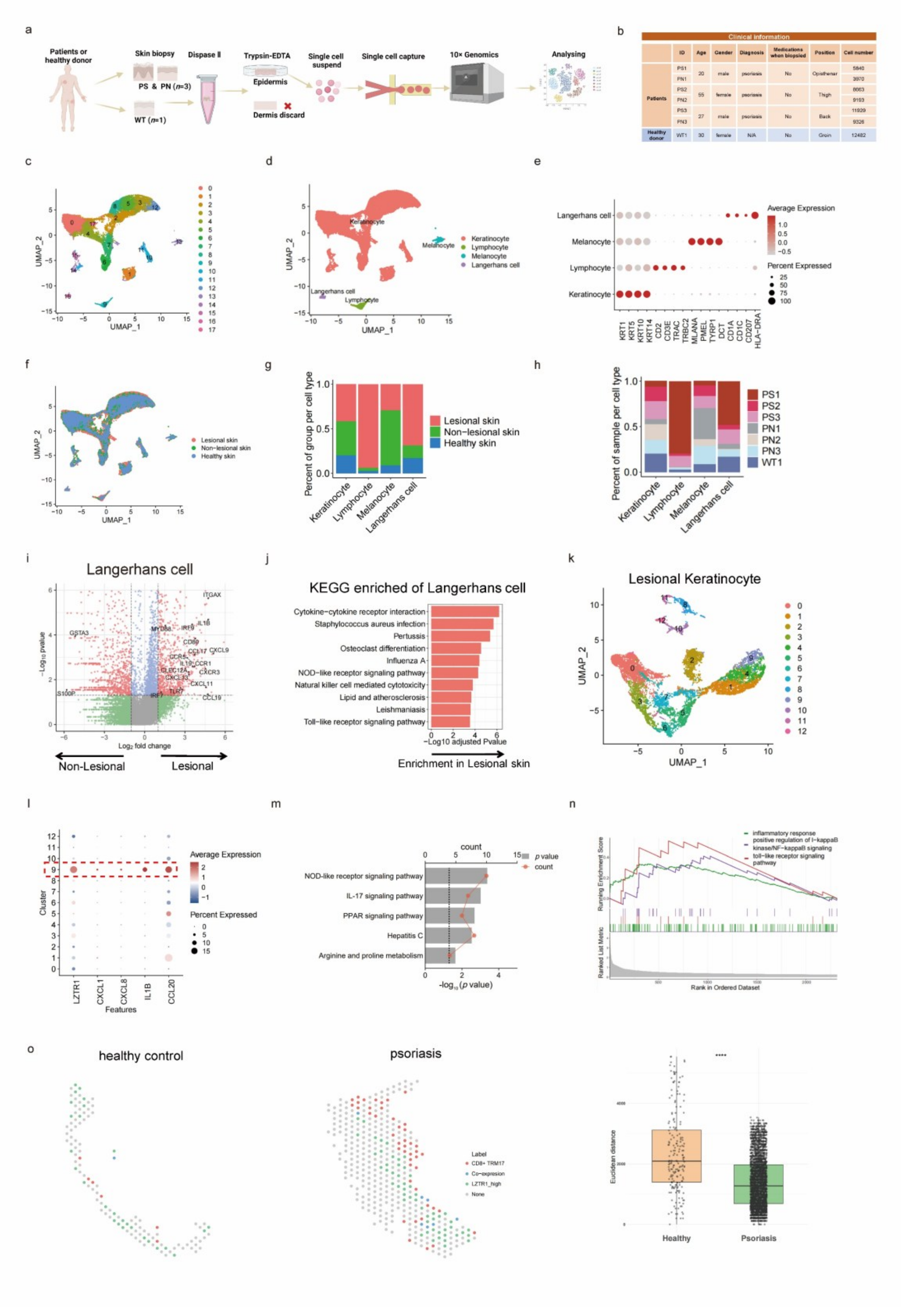

Supplementary Fig S1. ***LZTR1^{high}*** **KCs show hallmark of psoriasis.**

(a) Workflow diagram of scRNA-seq from biopsy of paired lesional and non-lesional skin tissues from 3 patients and normal skin tissues from 1 healthy donor. PS, psoriasis lesional skin; PN, perilesional skin; WT, healthy skin. **(b)** Clinical information and number of cells sequenced in scRNA-seq analysis for all donors. **(c)**

UMAP plot showing epidermal global cells colored by clusters. **(d)** UMAP plot showing global cells colored by annotated cell types. **(e)** Dot plot showing signature genes enriched in 4 cell types. **(f)** UMAP plot showing global cells colored by different sample groups. **(g)** Bar plot showing the composition across the disease conditions for each cell type. **(h)** Bar plot showing the composition across the different samples for each cell type. **(i)** Volcano plot showing the DEGs of Langerhans cells between lesional and non-lesional group. **(j)** Bar plot of the top KEGG pathways enriched among DEGs in lesional versus non-lesional Langerhans cells. **(k)** UMAP plot showing KCs colored by sub-clusters. **(l)** Dot plot illustrating the mRNA expression levels of *LZTR1* and several proinflammatory factors in each KC cluster from psoriasis lesional skin by scRNA-seq. **(m)** Enrichment on marker genes in Cluster 9 according to the KEGG database. **(n)** GSEA of genes related to biological process in Cluster 9. **(o)** Euclidean distances between $CD8^{+}$ T_{RM17} spots and LZTR1-high spots in healthy and psoriasis lesions. ****$P < 0.0001$ by Student's t-test.

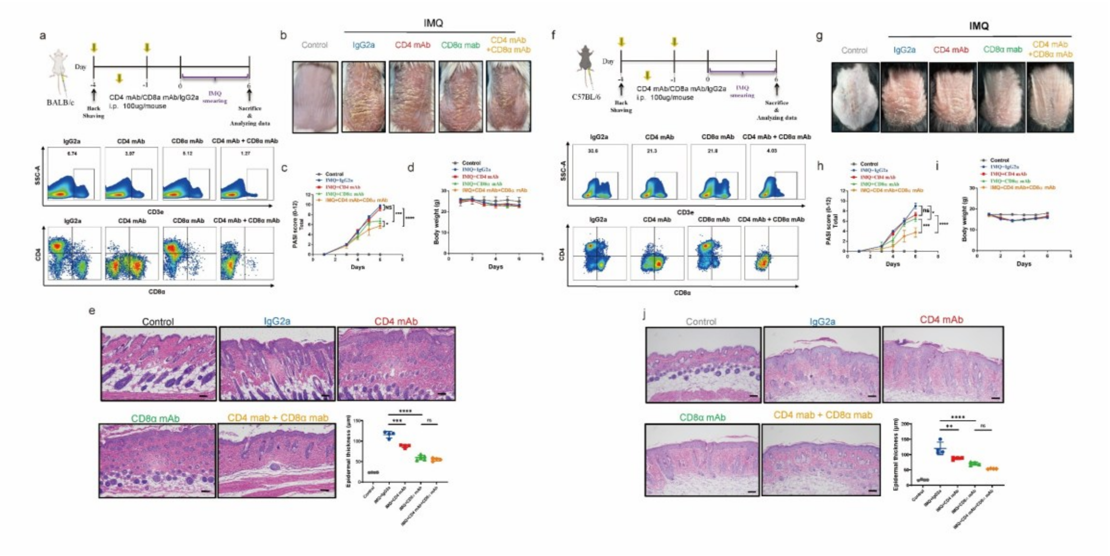

Supplementary Fig S2. CD8$^+$ T is indispensable for psoriatic pathogenesis.

(a) Experimental workflow and efficiency examination for CD4$^+$ or CD8$^+$ T depletion in BALB/c. **(b to e)** Macroscopic views **(b)**, PASI score **(c)**, representative H&E images and quantification of epidermal thickness of the back skin from BALB/c at the end point of study **(e)** and body weight of BALB/c during IMQ induction **(d)** (n=4-5). Scale bars, 100 μm. **(f)** Experimental workflow and efficiency examination for CD4$^+$ or CD8$^+$ T depletion in C57BL/6. **(g to j)** Macroscopic views **(g)**, PASI score **(h)**, representative H&E images, and quantification of epidermal thickness of the back skin from C57BL/6 at the end point of the study **(j)** and body weight of C57BL/6 during IMQ induction **(i)** (n=4-5). Scale bars, 100 μm.

ns: not significant; $^*P < 0.05$, $^{**}P < 0.01$, $^{***}P < 0.001$, $^{****}P < 0.0001$ by one-way ANOVA **(e and j)** and two-way ANOVA **(c and h)**. Data are shown as mean ± SEM.

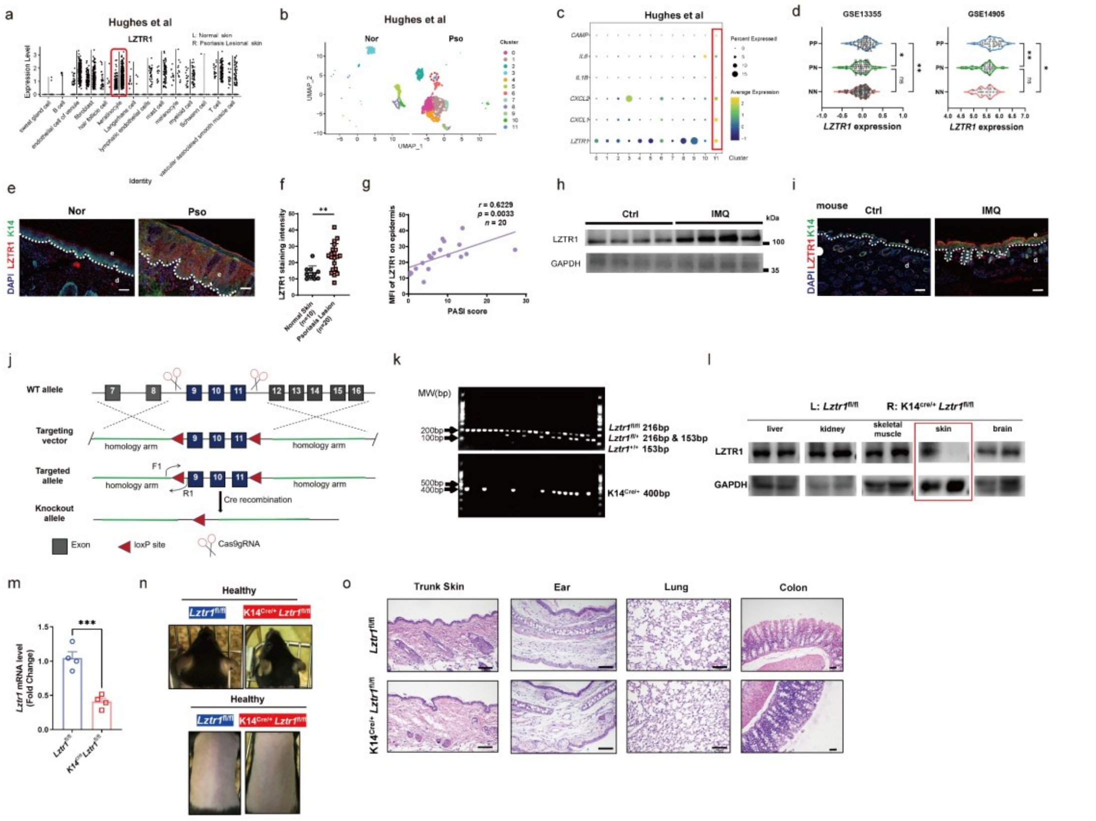

Supplementary Fig S3. Identification and characterization of LZTR1 in psoriatic KCs.

(a) Expression of *LZTR1* mRNA on different cell types reanalyzed from a public psoriasis scRNA-seq database (GSE150672) (n=8 psoriasis, n=3 healthy controls). **(b)** UMAP of KCs from lesional skin. Nor: normal, Pso: psoriasis. **(c)** Dot plot showing the expressions of *LZTR1* and psoriasis-related genes in each KC cluster. **(d)** The mRNA level of *LZTR1* was compared between psoriasis lesional skin (PP) and non-lesional skin (PN), or normal skin from controls (NN) in two public databases (GSE13355, GSE14905). **(e)** IF labeling of LZTR1 and K14 in human psoriasis lesion (n=20) and normal skin (n=10). e, epidermis; d, dermis. Scale bars, 100 μm. **(f)** The statistic results of LZTR1 staining intensity in two groups. **(g)** Linear regression analysis of MFI of LZTR1 and PASI score of individual patients (n=20). **(h)** IB analysis of LZTR1 expression in epidermis derived from mice treated with imiquimod (IMQ) or vehicle (Ctrl) (n=4). **(i)** IF images of back skin stained with K14, LZTR1, and DAPI. e, epidermis; d, dermis. Scale bars, 100 μm. **(j)** Schematic design of sgRNA targeting the *Lztr1* loci. **(k)** Genotyping analysis of WT (+/+), heterozygous (fl/+), and homozygous (fl/fl) transgenic mice using specific primers. **(l)** Immunoblotting analysis of LZTR1 knockout efficiency in different organs of *Lztr1*$^{fl/fl}$ (left lane) and K14$^{Cre/+}$ *Lztr1*$^{fl/fl}$ mice (right lane). **(m)** qPCR of *Lztr1* mRNA expression in mouse skin (n=4). **(n)** Representative images of ear and dorsal skin from *Lztr1*$^{fl/fl}$ and K14$^{Cre/+}$ *Lztr1*$^{fl/fl}$ mice. **(o)** Representative images of trunk skin, ear,

lung, and colon stained by H&E from *Lztr1*$^{fl/fl}$ and K14$^{Cre/+}$ *Lztr1*$^{fl/fl}$ mice. Scale bars, 100 μm.

ns: not significant; *$P < 0.05$, **$P < 0.01$, ***$P < 0.001$ by two-tailed unpaired t-test **(f and m)**, and one-way ANOVA **(d)**. Data are shown as mean ± SEM.

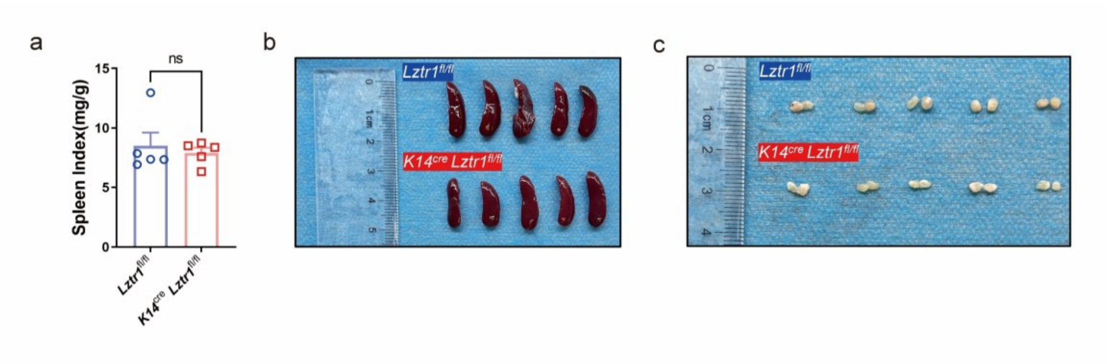

Supplementary Fig S4. Mice lacking *Lztr1* in the epidermis show no change in spleen and lymph node morphology.

(a) Spleen index of mice after psoriasis model (*n*=5). **(b and c)** The images of spleen **(b)** and inguinal lymph node **(c)** from *Lztr1*$^{fl/fl}$ and K14$^{Cre/+}$ *Lztr1*$^{fl/fl}$ mice.
ns: not significant; by two-tailed unpaired t-test **(a)**. Data are shown as mean ± SEM.

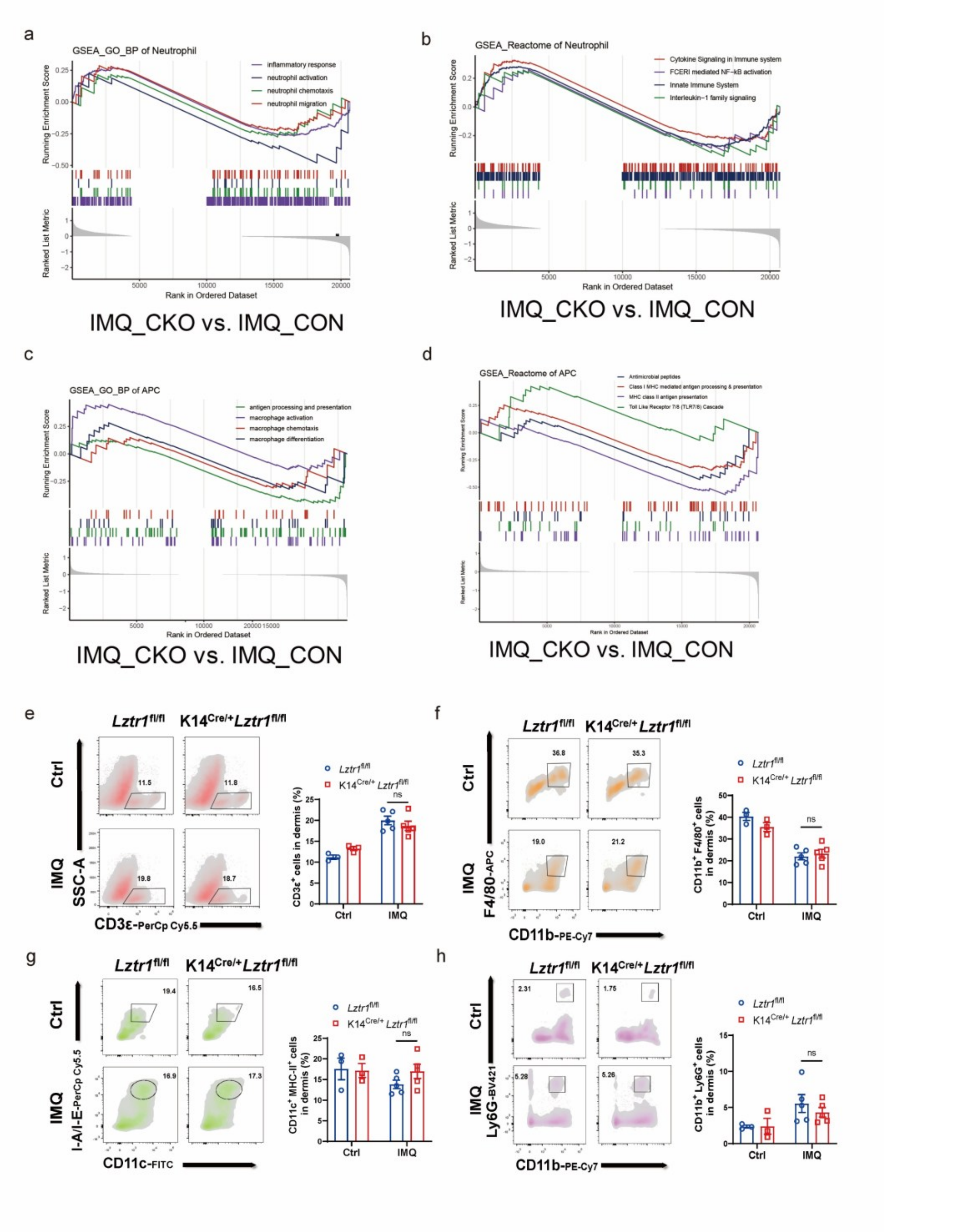

Supplementary Fig S5. Genetic deletion of *Lztr1* has no impact on innate immune cells.

(a-d) Enriched pathways on GO and Reactome of neutrophil **(a, b)** and APC **(c, d)** by GSEA analysis. BP, Biological Process. **(e-h)** Flow cytometry analysis on the cell proportion of T cells **(e)**, macrophages **(f)**, dendritic cells **(g)**, and neutrophils **(h)** in mouse skin lesion with or without IMQ treatment. Right plot, quantification (n=3-5). ns: not significant by two-tailed unpaired t-test **(e-h)**. Data are shown as mean ± SEM.

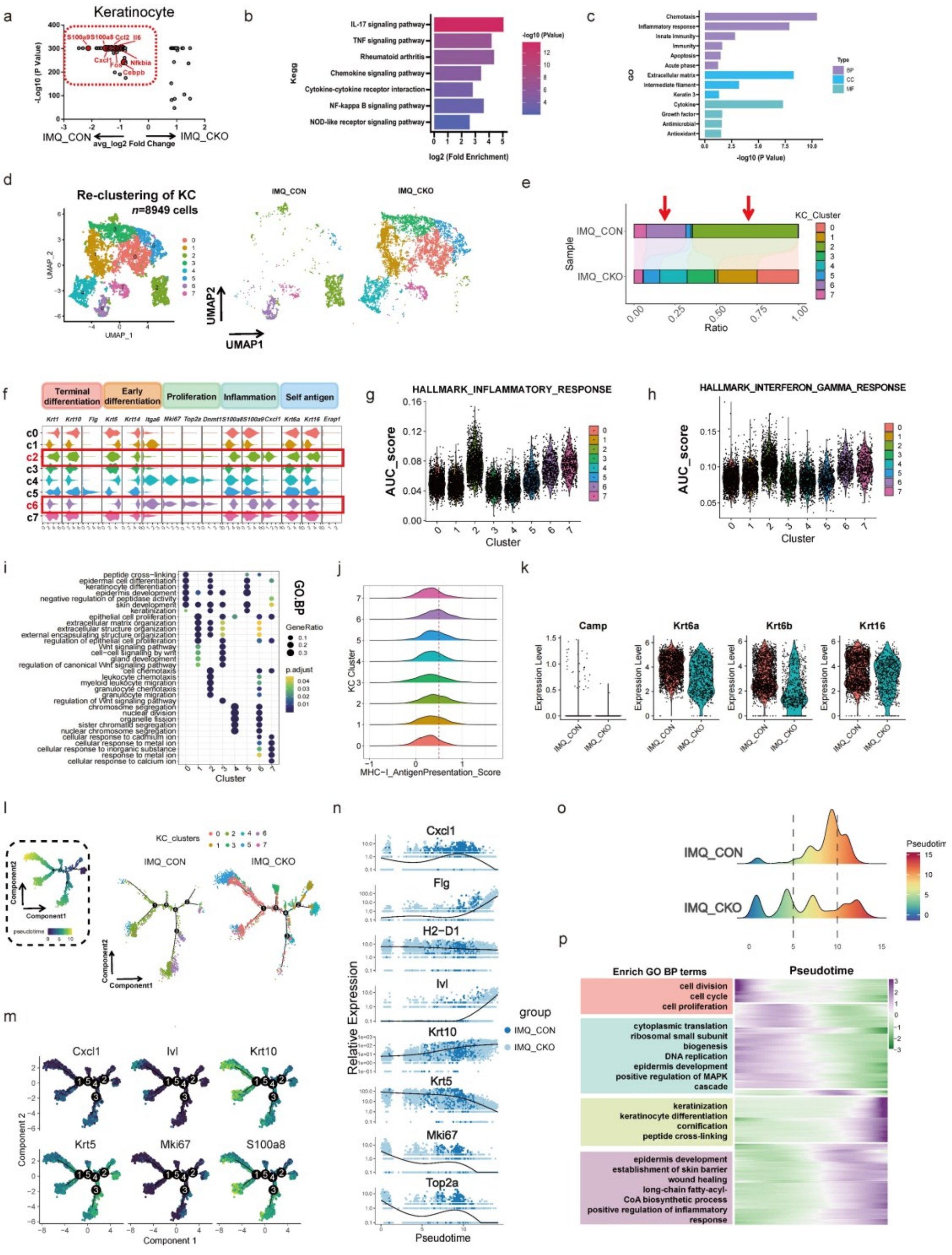

Supplementary Fig S6. LZTR1 modulates KC inflammatory responses and keratinization in inflamed skin.

(a) Volcano plots comparing DEGs in KCs between IMQ_CKO and IMQ_CON group from scRNA-seq. **(b, c)** KEGG **(b)** and GO **(c)** analysis of downregulated genes enriched in KC from CKO mice ($log_2FC < -1$ & $P < 0.05$). **(d)** UMAP plot of subcluster in KCs. **(e)** The proportion of different KC subclusters among samples. **(f)** Violin plot showing the representative marker genes of KC subcluster. **(g, h)** Violin

plot showing enriched hallmark AUC score of each KC subcluster. **(i)** Dot plot showing GO biological process terms enriched in KC subcluster marker genes. **(j)** The distribution of MHC-I antigen presentation score of KCs in different subclusters. **(k)** The expression level of potential self-antigen in top three MHC-I antigen presentation score KC subclusters (cluster 1, 2, 6) among different samples. **(l)** Trajectory analysis of KCs among two groups. **(m, n)** Expression of selected genes along pseudotime. **(o)** KCs distribution density along pseudotime. **(p)** Heatmap showing relative expression of genes significantly varying with pseudotime. Relevant GO terms are highlighted.

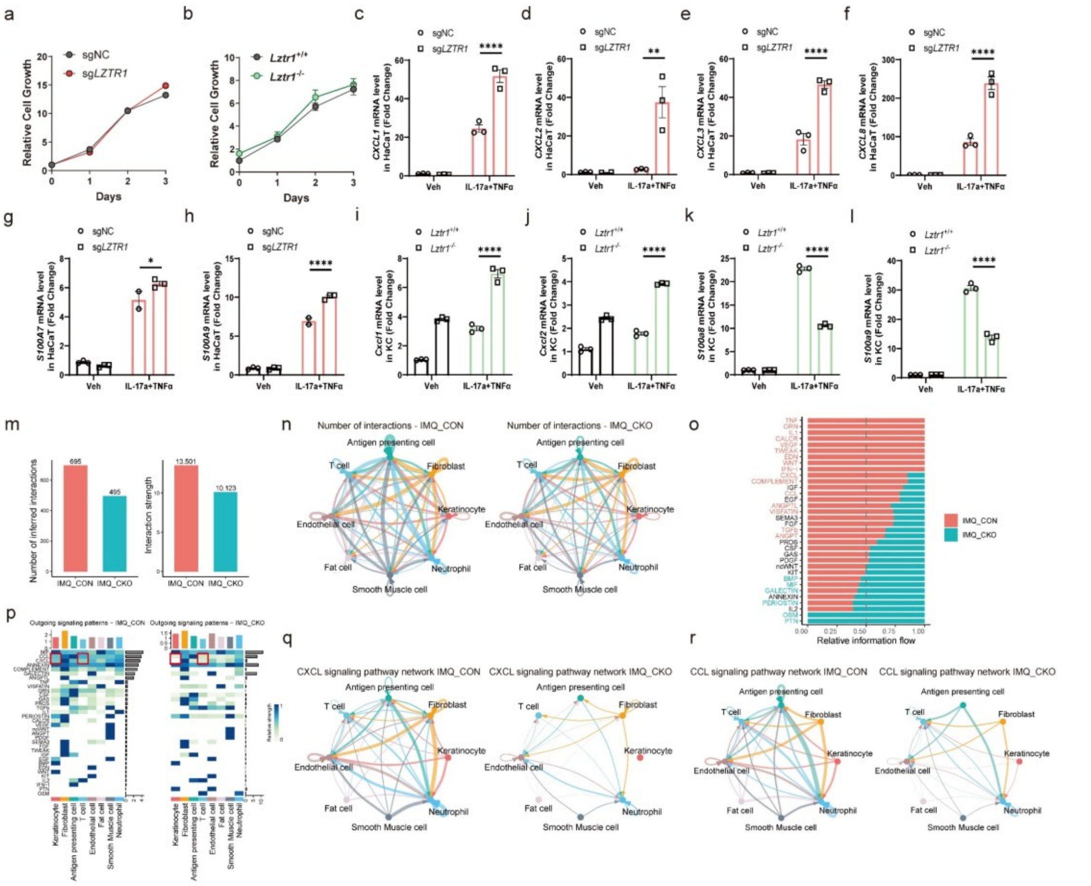

Supplementary Fig S7. Suppression on KC-T cell interaction is responsible for the ameliorated psoriasis phenotype.

(a, b) CCK-8 assay analysis of relative cell proliferation of HaCaT **(a)** or primary mouse KC **(b)**, and the ratio of OD value from end point to start point was calculated and plotted (n=6). **(c-h)** qPCR of *CXCL1, CXCL2, CXCL3, CXCL8, S100A7, S100A9* in sgNC and sg*LZTR1* HaCaT cells treated with or without IL17A and TNF-α for 24 h (n=2-3). **(i-l)** qPCR of *Cxcl1, Cxcl2, S100a8, S100a9* in primary mouse KCs extracted from *Lztr1*$^{fl/fl}$ and K14$^{Cre/+}$ *Lztr1*$^{fl/fl}$ mice treated with or without IL17A and TNF-α (n=3). **(m)** Bar plot of inferred interactions and interaction strength in CON and CKO group. **(n)** Cell-cell interactions in IMQ_CON and IMQ_CKO group, the link size represents the interaction strengthen. **(o)** Relative information flow of cytokines in the CKO group compared to the CON group. **(p)** Heatmap showing the release of CCL and CXCL chemokine signaling from different cell types. **(q, r)** The CXCL **(q)** and CCL **(r)** signaling pathway network showing intercellular interaction between IMQ_CON and IMQ_CKO group.

ns: not significant; *$P < 0.05$, **$P < 0.01$, ****$P < 0.0001$ by two-way ANOVA **(c-l)**. Data are shown as mean ± SEM.

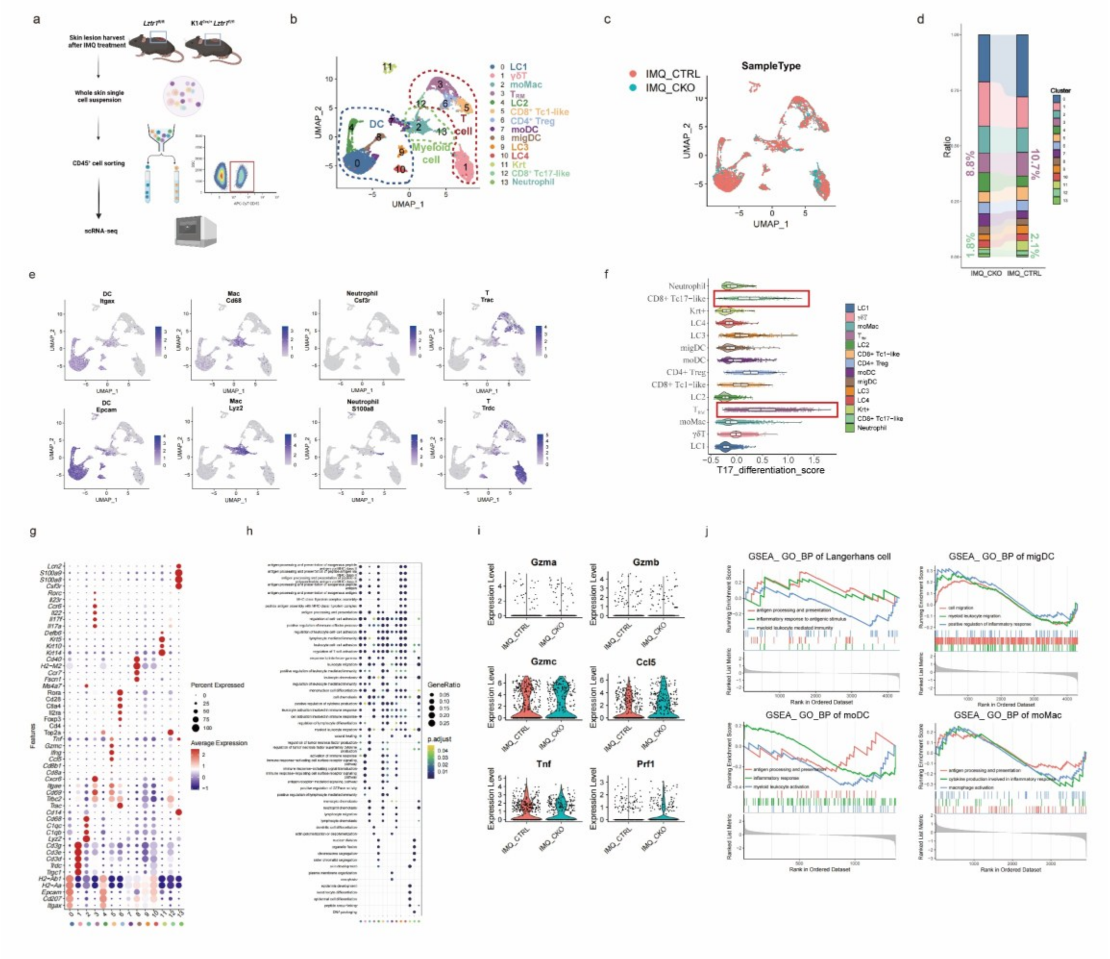

Supplementary Fig S8. scRNA-seq analysis exhibits immune cells from psoriasis-like mouse lesional tissues after CD45$^+$ sorting.

(a) Overview of the workflow for the analysis of CD45$^+$ immune cells from IMQ-treated skin for scRNA-seq analysis. **(b and c)** UMAP projection of 14 clusters of skin immune cells **(b)** and projection between two groups **(c)**. **(d)** Proportion of different subtype immune cells. **(e)** Feature plot of signature genes enriched in 4 types of immune cells. **(f)** Violin plots showing T17 differentiation score in each cluster. **(g)** Dot plot displaying relative marker expression level among identified cell populations in scRNA-seq after CD45$^+$ sorting. **(h)** GO biological process enrichment analysis of function on 13 clusters. **(i)** The expression level of cytotoxic related genes in CD8$^+$ Tc1-like cells among different samples. **(j)** Enriched pathways on GO of Langerhans cells, migDC, moDC, and moMac cells by GSEA analysis.

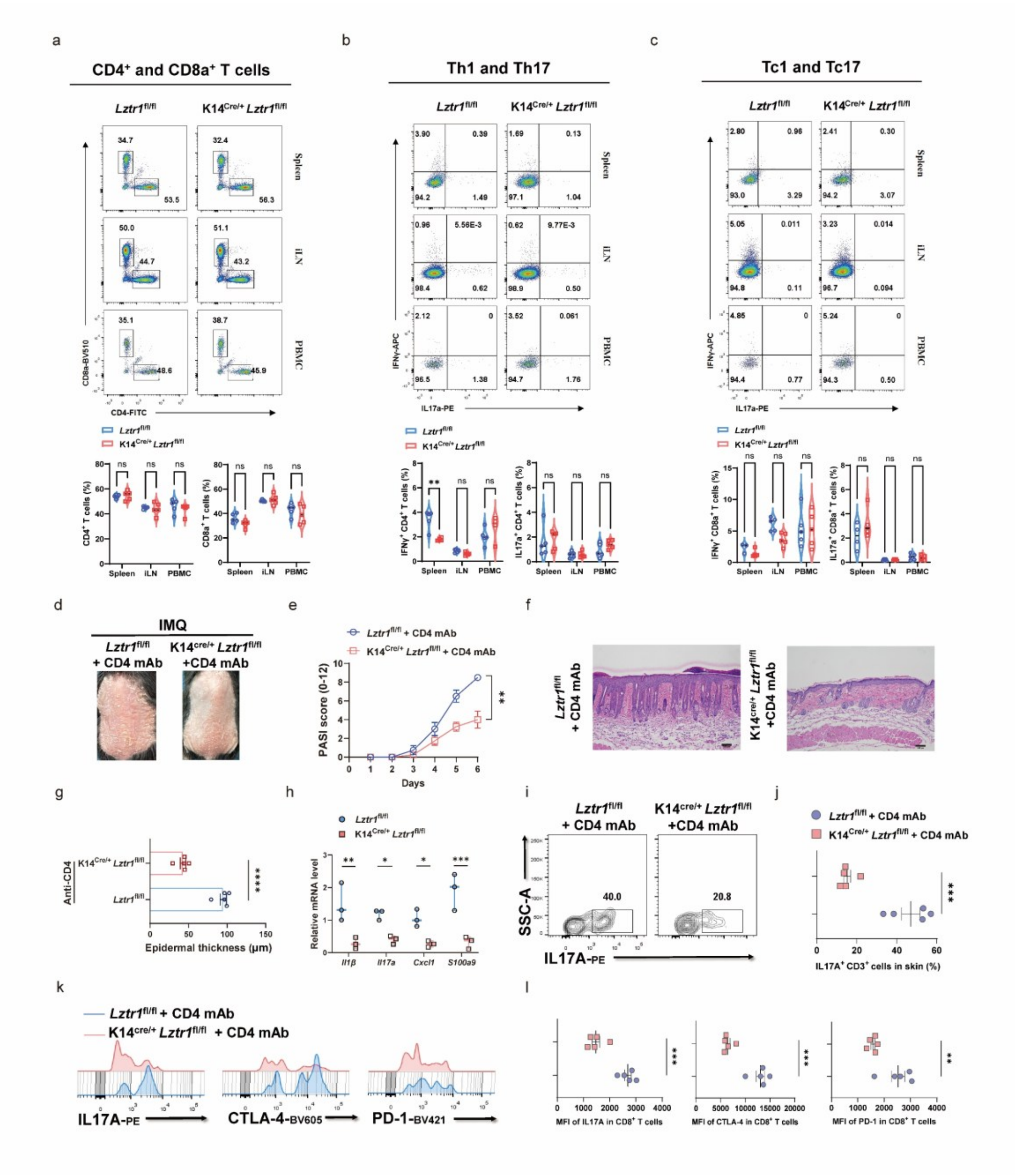

Supplementary Fig S9. *Lztr1*-deficient mice relieve skin inflammation independent of peripheral T cell and dermal CD4$^+$ T cells.

(a-c) Flow cytometry quantification and statistical chart of CD4$^+$ T and CD8$^+$ T cells **(a)**, Th1 and Th17 **(b)**, Tc1 and Tc17 **(c)** from spleen, iLN and PBMC (n=3-5). **(d, e)** Results of lesional skin images **(d)**, PASI score variation chart **(e)** (n=5). **(f, g)** H&E staining of skin biopsies, scale bars, 100 μm **(f)**, and measurement data for epidermal thickness **(g)** (n=5). **(h)** qPCR analysis of mRNA level of *Il1β, Il17a, Cxcl1, S100a9* in skin lesions (n=3). **(i, j)** Flow cytometry **(i)** and statistical chart **(j)** of IL-17A$^+$ among CD3e$^+$ cells in skin (n=5). **(k, l)** Univariate histogram of flow cytometry **(k)** and quantification **(l)** of MFI of IL-17A, CTLA-4, PD-1 in CD8$^+$ T cells (n=5).

ns: not significant; $*P < 0.05$, $**P < 0.01$, $***P < 0.001$, $****P < 0.0001$ by two-tailed unpaired t-test **(g, h, j and l)** and two-way ANOVA **(a-c and e)**. Data are shown as

mean ± SEM.

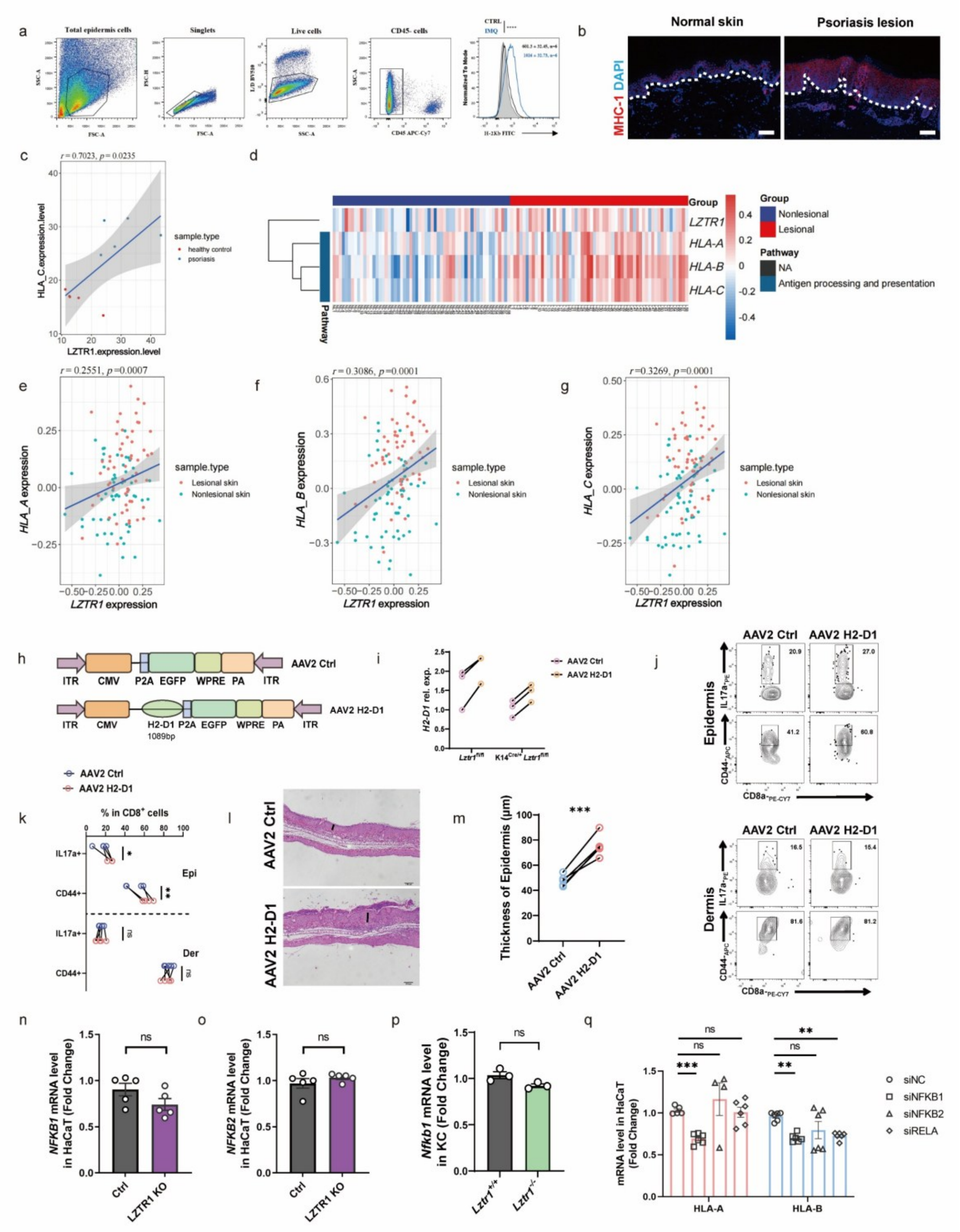

Supplementary Fig S10. Excessive epidermal MHC-I expression exacerbates psoriasis inflammatory response.

(a) Flow cytometry analysis of H-2K^{b} expression in epidermal CD45^{-} KCs from BALB/c mice treated with or without IMQ (n=6). **(b)** IF analysis of MHC-I expression in psoriasis lesion and healthy control (n=4-5). Scale bars, 100 μm. **(c)** Linear regression analysis of HLA-C and LZTR1 IF staining intensity between healthy control and patients with psoriasis. **(d)** Heatmap displaying relative expression level of *LZTR1*, *HLA-A*, *HLA-B*, and *HLA-C* in psoriasis lesional skin and

non-lesional skin. Data was derived from GSE13355. **(e-g)** Linear regression analysis of *LZTR1* mRNA expression and *HLA-A* **(e)**, *HLA-B* **(f)**, *HLA-C* **(g)** mRNA expression from psoriasis lesional and non-lesional skin. Data was derived from GSE13355. **(h)** Schematic design of AAV2 control vector and AAV2 H2-D1. **(i)** qPCR analysis of *H2-D1* mRNA expression in mice ear skin tissue 7 days after AAV2 intracutaneous injection. **(j)** Flow cytometry analysis on frequencies of IL17A$^+$ or CD44$^+$ cells of CD8$^+$ T in epidermis and dermis. **(k)** Corresponding quantification (n=4-5). **(l)** H&E staining of ear skin sections on day 6 after IMQ treatment. Scale bars, 100 µm. **(m)** Summary of epidermal thickness (n=4). **(n, o)** qPCR analysis of *NFKB1* **(n)** and *NFKB2* **(o)** mRNA expression in Ctrl or *LZTR1* KO HaCaT cells (n=5). **(p)** qPCR analysis of *Nfkb1* expression in primary KCs from *Lztr1*$^{fl/fl}$ and K14$^{Cre/+}$ *Lztr1*$^{fl/fl}$ mice (n=3). **(q)** qPCR analysis of *HLA-A* and *HLA-B* mRNA expression in HaCaT cells treated with siNC, si*NFKB1,* si*NFKB2,* si*RELA* interference, respectively (n=4-6).
ns: not significant; **P* < 0.05, ***P* < 0.01, ****P* < 0.001, *****P* < 0.0001 by two-tailed paired t-test **(k, m)**, two-tailed unpaired t-test **(a and n-p)** and one-way ANOVA **(q)**. Data are shown as mean ± SEM.

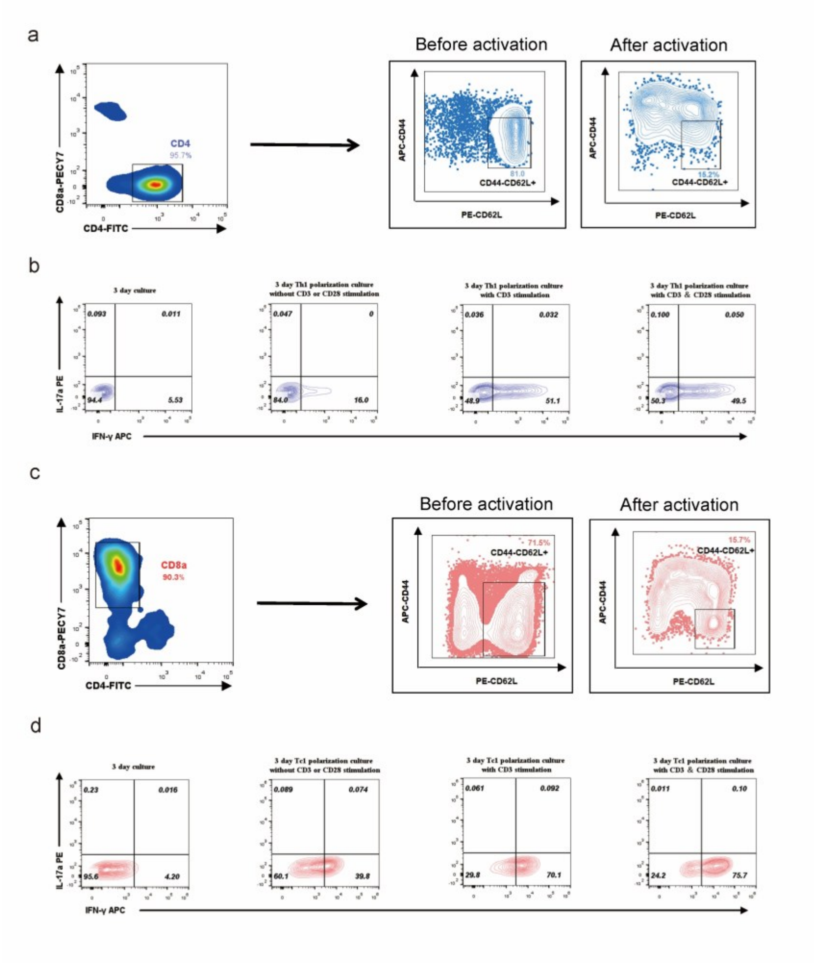

Supplementary Fig S11. Continuous first signal is indispensable for activated CD4$^+$ T and CD8$^+$ T cells to get complete differentiation.

(**a**) Left, flow cytometry detecting efficiency of mouse primary CD4$^+$ T cells isolation. Right, flow cytometry detecting mature and naive status of CD4$^+$ T cells before or after 72 h stimulation with anti-CD3/CD28. **(b)** After 72 h stimulation with anti-CD3/CD28, CD4$^+$ T cells were collected and given following treatment for another 3 days: first group, IL2 (20 ng/mL) + anti-IL4 (10 μg/mL); second group, IL2 (20 ng/mL) + anti-IL4 (10 μg/mL) + IL12 (20 ng/mL); third group, IL2 (20 ng/mL) + anti-IL4 (10 μg/mL) + IL12 (20 ng/mL) + anti-CD3e (5 μg/mL); forth group, IL2 (20 ng/mL) + anti-IL4 (10 μg/mL) + IL12 (20 ng/mL) + anti-CD3e (5 μg/mL) + anti-CD28 (2 μg/mL). Flow cytometry detecting IFNγ expression of CD4$^+$ T cells (Th1 differentiation). **(c)** Left, flow cytometry detecting efficiency of mouse primary CD8$^+$ T cells isolation. Right, flow cytometry detecting mature and naive status of CD8$^+$ T cells before or after 72 h stimulation with anti-CD3/CD28. **(d)** After 72 h stimulation with anti-CD3/CD28, CD8$^+$ T cells were collected and given following treatment for another 3 days: first group, IL2 (20 ng/mL) + anti-IL4 (10 μg/mL); second group, IL2 (20 ng/mL) + anti-IL4 (10 μg/mL) + IL12 (20 ng/mL); third group, IL2 (20 ng/mL) + anti-IL4 (10 μg/mL) + IL12 (20 ng/mL) + anti-CD3e (5 μg/mL); forth group, IL2 (20 ng/mL) + anti-IL4 (10 μg/mL) + IL12 (20 ng/mL) + anti-CD3e (5 μg/mL) + anti-

CD28 (2 μg/mL). Flow cytometry detecting IFNγ expression of $CD8^+$ T cells (Tc1 differentiation).

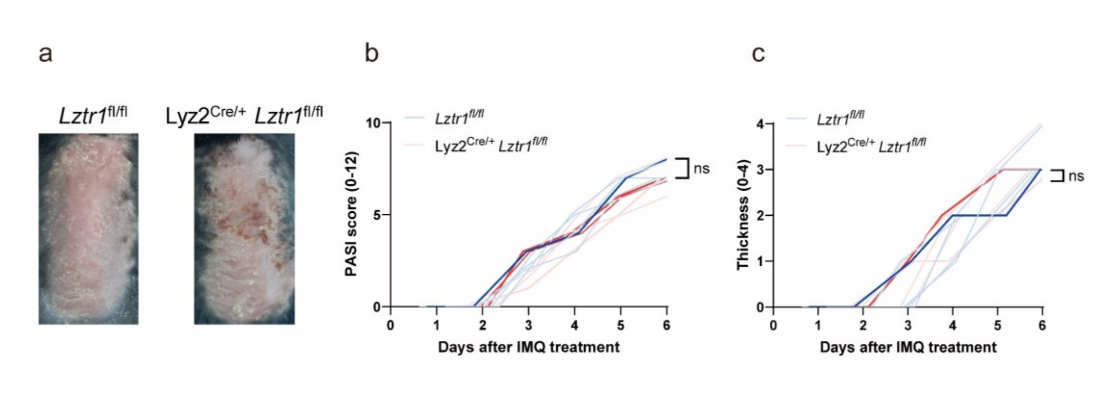

Supplementary Fig S12. Lyz2$^{Cre/+}$ *Lztr1*$^{fl/fl}$ mice have no amelioration on psoriasis prognosis.

(a) Photos of back skin in *Lztr1*$^{fl/fl}$ and Lyz2$^{Cre/+}$ *Lztr1*$^{fl/fl}$ mice treated with IMQ for 6 days. **(b)** PASI score (0-12) of back skin in *Lztr1*$^{fl/fl}$ and Lyz2$^{Cre/+}$ *Lztr1*$^{fl/fl}$ mice. **(c)** Thickness score (0-4) of back skin in *Lztr1*$^{fl/fl}$ and Lyz2$^{Cre/+}$ *Lztr1*$^{fl/fl}$ mice.

ns: not significant by two-way ANOVA **(b and c)**. Data are shown as mean ± SEM.

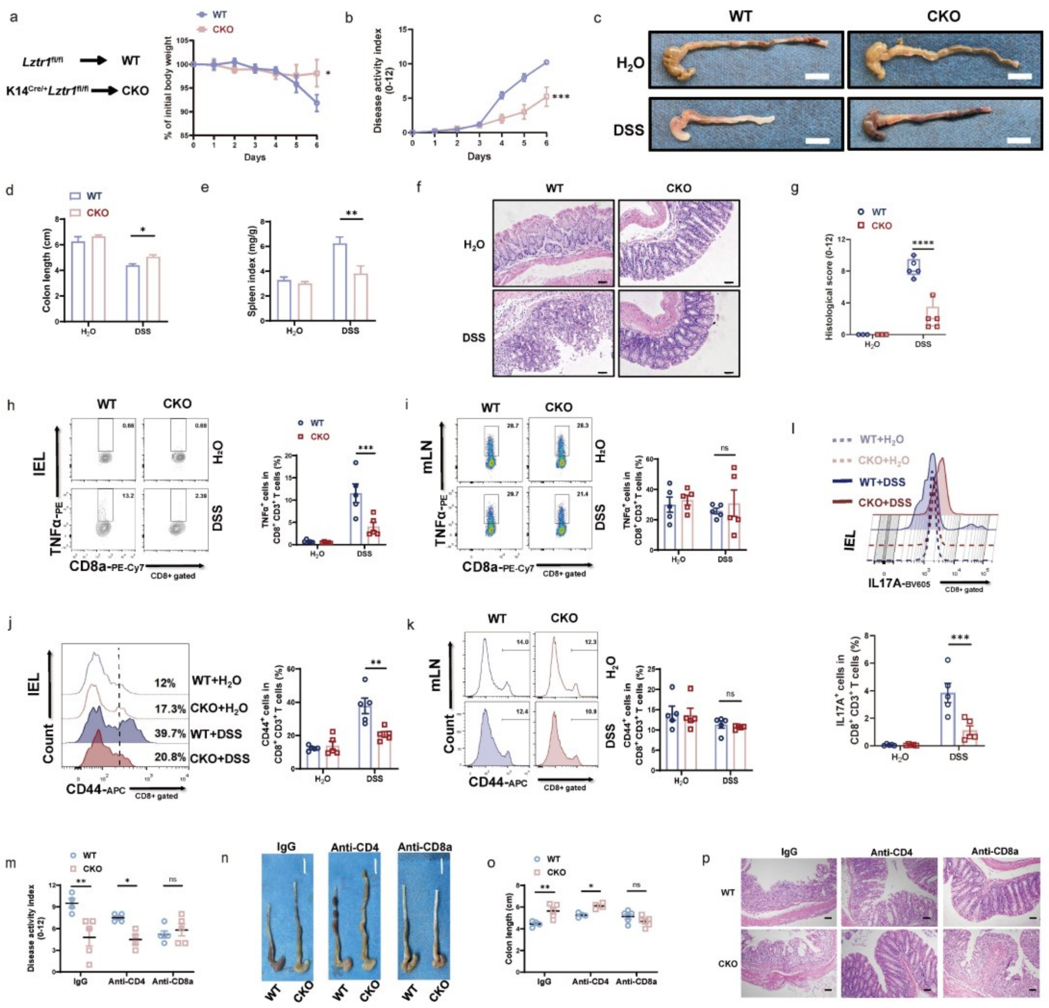

Supplementary Fig S13. Contribution of LZTR1 to CD8$^+$ T cell crosstalk in gastrointestinal epithelium in the DSS colitis model of IBD.

(a) Weight changes are analyzed as the mean change from the initial weight (n=5). **(b)** DAI change (n=5). **(c)** Representative photographs of colons from WT and CKO mice treated with DSS or drinking water for 6 days. Scale bars, 1cm. **(d and e)** Colon length **(d)** and spleen index **(e)** were calculated (n=3-5). **(f and g)** Representative H&E images **(f)** and corresponding histological score of colon tissues **(g)** (n=3-5). Scale bars, 100 µm. **(h and i)** Flow cytometry showing the percentage of TNFα$^+$ among CD3$^+$ CD8$^+$ T cells from intraepithelial lymphocyte (IEL) **(h)** and mesenteric lymph nodes (mLN) **(i)** between WT and CKO mice treated with DSS or drinking water (n=5). **(j and k)** Flow cytometry showing the percentage of CD44$^+$ among CD3$^+$ CD8$^+$ T cells from IEL **(j)** and mLN **(k)** between WT and CKO mice treated with DSS or drinking water (n=5). **(l)** Flow cytometry showing the percentage of IL-17A among CD3$^+$ CD8$^+$ T cells from IEL (up) between WT and CKO mice treated with DSS or drinking water, and corresponding quantification (down) (n=5). **(m to p)** DAI score **(m)**, representative images of colon **(n)**, scale bars, 1 cm, colon length

statistic **(o)** and H&E image **(p)** from WT and CKO mice after DSS induction upon $CD4^+$ or $CD8^+$ T cell deletion (n=4-5). Scale bars, 100 μm.
ns: not significant; *$P < 0.05$, **$P < 0.01$, ***$P < 0.001$, ****$P < 0.0001$ by two-way ANOVA **(a, b, d, e, g-m and o)**. Data are shown as mean ± SEM.

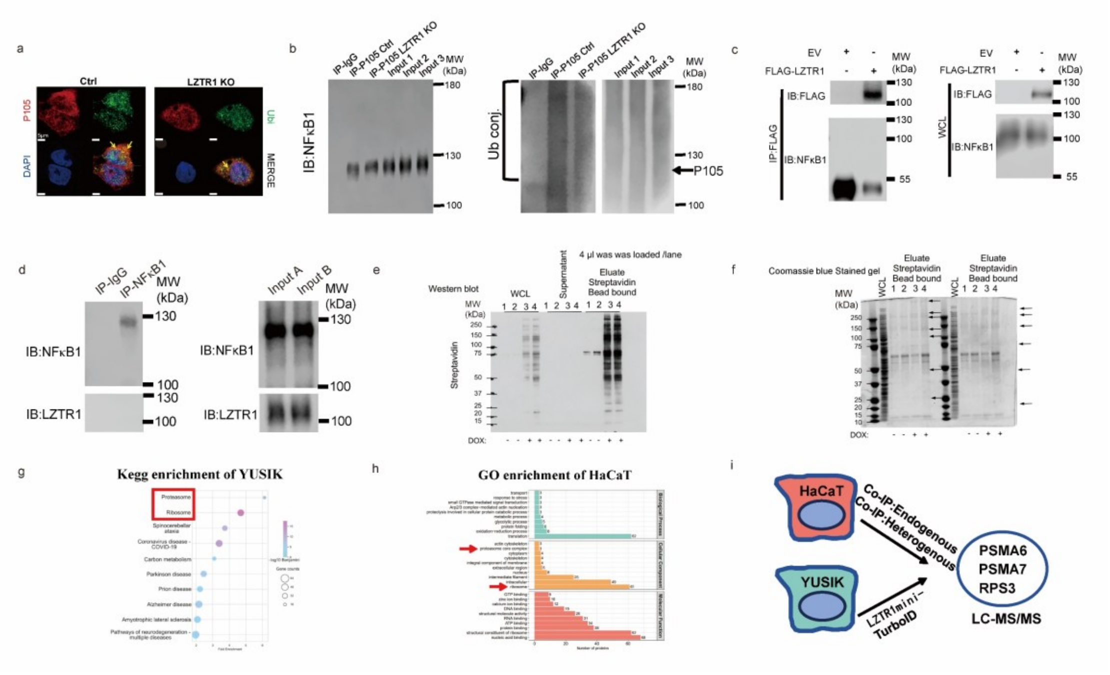

Supplementary Fig S14. LZTR1 regulates NF-κB1 P50 generation without directly affecting NF-κB1 p105 ubiquitination process.

(**a**) IF of NF-κB1 (P105, red) and Ubiquitin (Ubi, green) in *LZTR1* knockout or control HaCaT cells by confocal microscopy. Scale bars, 5 μm. **(b)** IB analysis of NF-κB1 (left) and Ubiquitin conjunction (right) in P105 precipitates immunoprecipitated from sgNC or sg*LZTR1* HaCaT cells. Input 1-3 (lanes 4-6) represent 10% of respective precleared cell lysates of lanes 1-3. **(c)** IB analysis of FLAG-LZTR1 and NF-κB1 in FLAG-LZTR1 precipitates immunoprecipitated from HaCaT cells reconstituted with EV or FLAG-tagged human LZTR1. **(d)** IB analysis of NF-κB1 and LZTR1 in NF-κB1 precipitates immunoprecipitated from wild type HaCaT cells. **(e)** IB analysis of biotinylated proteins bound to streptavidin beads. WCL, whole cell lysates. DOX, doxycycline. **(f)** Coomassie blue staining analysis of biotinylated proteins bound to streptavidin beads. Arrows on the right exhibit potential interaction proteins weight. DOX, doxycycline. **(g)** Kegg enrichment of interacted proteins of LZTR1 from proteomics in YUSIK transfected with pIND-LZTR1-HA-miniTurboID plasmid. **(h)** GO enrichment of interacted proteins of LZTR1 from proteomics in HaCaT expressed exogenously LZTR1-FLAG. **(i)** Schematic diagram of the mutual interacted proteins related with proteasome and ribosome among two different cell types according to cell proteomics from YUSIK and HaCaT cells.

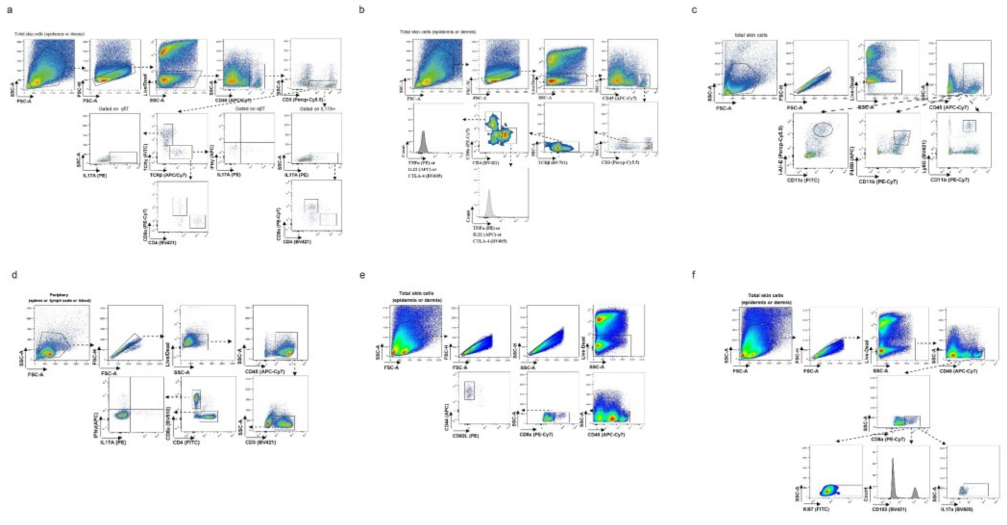

Supplementary Fig S15. Flow cytometry gating strategies.

(a and b) Gating strategies of different types of T cells from skin. **(c)** Gating strategies of dendritic cell, macrophage, and neutrophil from skin. **(d)** Gating strategies of different types of T cells from spleen, lymph node or PBMC. **(e)** Gating strategies of $CD8^+$ $CD44^+$ $CD62L^-$ effector memory T cells. **(f)** Gating strategies of Ki67, CD103, IL-17A expression of $CD8^+$ T cells.

Supplementary Table S1. Related Sequences Table

qPCR primer nucleotide sequences			
**Gene**	**Species**	**Forward primer**	**Reverse primer**
Gapdh	mouse	AGGTCGGTGTGAACGGATTTG	TGTAGACCATGTAGTTGAGGTCA
Cxcl1	mouse	AGAACATCCAGAGCTTGAAGG	CAATTTTCTGAACCAAGGGAGC
Cxcl2	mouse	CCAACCACCAGGCTACAGG	GCGTCACACTCAAGCTCTG
Il1b	mouse	ACGGACCCCAAAAGATGAAG	TTCTCCACAGCCACAATGAG
Il6	mouse	CAAAGCCAGAGTCCTTCAGAG	GTCCTTAGCCACTCCTTCTG
S100a8	mouse	AGTGTCCTCAGTTTGTGCAG	ACTCCTTGTGGCTGTCTTTG
S100a9	mouse	ATACTCTAGGAAGGAAGGACACC	TCCATGATGTCATTTATGAGGGC
Il22	mouse	AGCTTGAGGTGTCCAACTTC	GGTAGCACTGATCCTTAGCACTG
Il17a	mouse	TCCAGAATGTGAAGGTCAACC	TATCAGGGTCTTCATTGCGG
Ccl20	mouse	AAGACAGATGGCCGATGAAG	TCTTGACTCTTAGGCTGAGGA
H2-d1	mouse	GAAGTGGGCATCTGTGGTGGTG	ATGTAAGAGTCAGTGGACGGAGGAG
H2-k1	mouse	ACCAGCAGTACGCCTACGA	AACCAGAACAGCAACGGTCG
Nfkb1	mouse	GGAGGCATGTTCGGTAGTGG	CCCTGCGTTGGATTTCGTG
Lztr1	mouse	TTGACAGGAGGGGTACGCT	ATATGCCACGACTGTGTGCTT
GAPDH	human	GGAGCGAGATCCCTCCAAAAT	GGCTGTTGTCATACTTCTCATGG
HLA-DRA	human	ATACTCCGATCACCAATGTACCT	GACTGTCTCTGACACTCCTGT
HLA-DRB	human	CGGGGTTGGTGAGAGCTTC	AACCACCTGACTTCAATGCTG
HLA-A	human	AAAAGGAGGGAGTTACACTCAGG	GCTGTGAGGGACACATCAGAG
HLA-B	human	CAGTTCGTGAGGTTCGACAG	CAGCCGTACATGCTCTGGA
HLA-C	human	TCTACCCTGCGGAGATCACACTG	GCTCTTGTCCAGAAGGCACCAC
NFKB1	human	AACAGAGAGGATTTCGTTTCCG	TTTGACCTGAGGGTAAGACTTCT
NFKB2	human	GGGCCGAAAGACCTATCCC	CAGCTCCGAGCATTGCTTG
RELA	human	GTGGGGACTACGACCTGAATG	GGGGCACGATTGTCAAAGATG
CXCL1	human	CCGAAGTCATAGCCACACTCAAG	GTTGGATTTGTCACTGTTCAGCATC
CXCL2	human	ATCCAAAGTGTGAAGGTGAAGTCC	AGCTTTCTGCCCATTCTTGAGTG
CXCL3	human	CCAAACCGAAGTCATAGCCAC	TGCTCCCCTTGTTCAGTATCT
CXCL8	human	TTTTGCCAAGGAGTGCTAAAGA	AACCCTCTGCACCCAGTTTTC
S100A8	human	ATGCCGTCTACAGGGATGAC	ACTGAGGACACTCGGTCTCTA
S100A9	human	GGTCATAGAACACATCATGGAGG	GGCCTGGCTTATGGTGGTG

Genotyping primer nucleotide sequences		
**Genotyping**	**Forward primer**	**Reverse primer**
Lztr1-flox	GCTGTCTATCCTTGGACCCTATG	AGAACTGTATTCTCTCTACCCAGGA
K14-cre	CGATGGGAAAGTGTAGCCTGCA	TCCAGGTATGCTCAGAAAACGCC
Lyz2-cre	CCCAGAAATGCCAGATTACG	CTTGGGCTGCCAGAATTTCTC
